# Supplementary material for: Exhausted CD8+ T cells exhibit low and strongly inhibited TCR signaling during chronic LCMV infection
Source: Nat Commun. 2020 Sep 8;11:4454. doi: 10.1038/s41467-020-18256-4 (PMC7479152; doi:10.1038/s41467-020-18256-4)
Supplement: Supplementary file 1 — Supplementary Information [file 41467_2020_18256_MOESM1_ESM.pdf]

# Exhausted CD8 T cells exhibit low and strongly inhibited TCR signaling during chronic LCMV infection

Sandu et al.

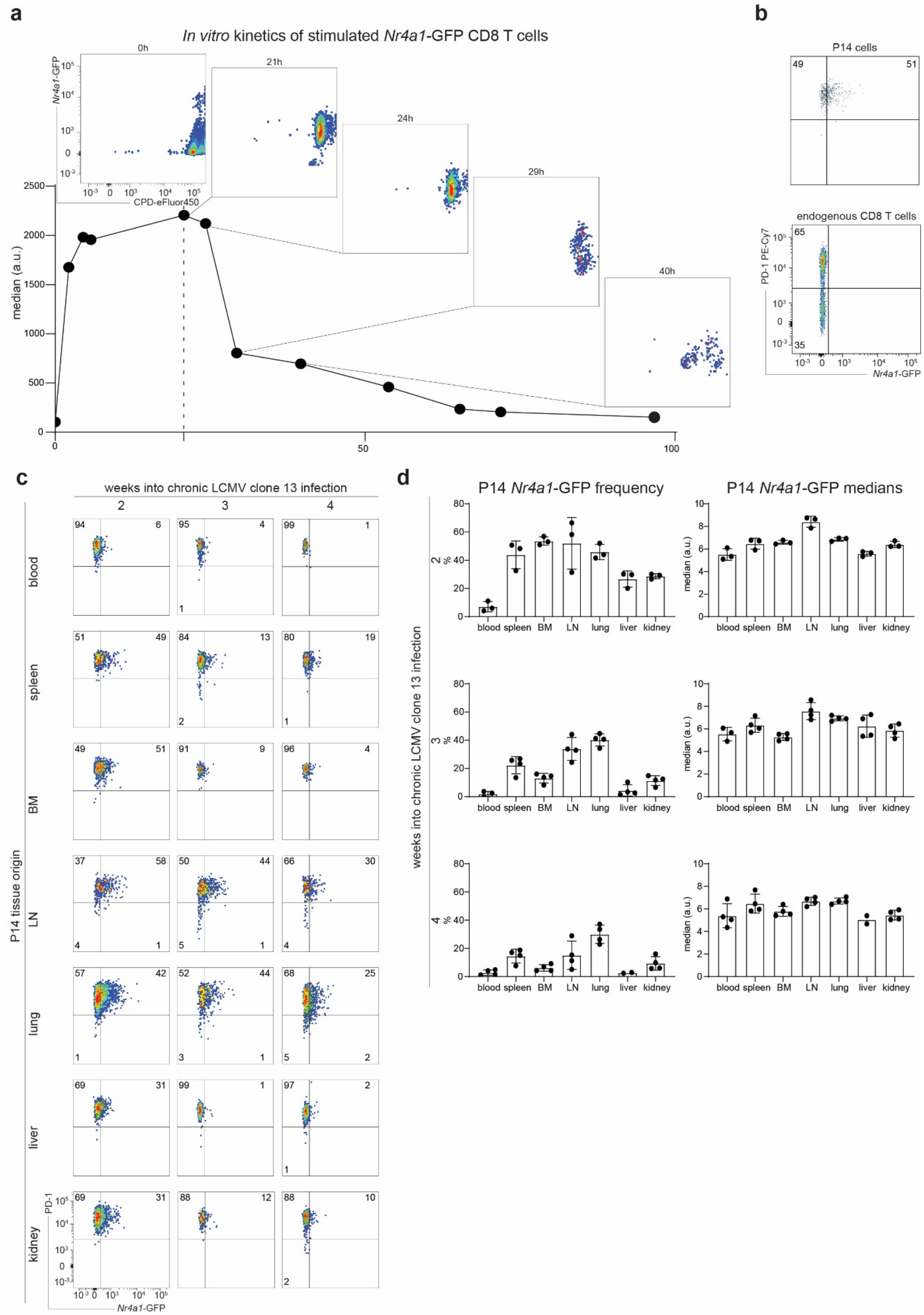


**Supplementary Fig. 1:** ***In vitro*** **kinetics of** ***Nr4a1*****-GFP after stimulation and** ***ex vivo*** **expression in P14 cells.**

**a** *Nr4a1*-GFP CD8 T cells labeled with CPD (cell proliferation dye) were in vitro stimulated with plate-bound anti-CD3ε and anti-CD28 for 20 hours. Dotted line indicates the time when the stimulus was removed. Boxes

show the profile of CDP and *Nr4a1*-GFP levels at the specified time-points after activation. Dots represent mean of normalized *Nr4a1*-GFP expression (n = 2 mice). **b-d** CD45.1$^+$ P14-*Nr4a1*-GFP cells were transferred into CD45.2$^+$ hosts one day prior infection with a high dose of LCMV clone 13. **b** Non-transgenic endogenous CD8 T cells were used to set the gates: example showing *Nr4a1*-GFP signal in non-transgenic endogenous CD8 cells (gated on alive single CD8$^+$ cells) or transferred transgenic P14 *Nr4a1*-GFP cells (gated on alive single CD8$^+$ CD45.1$^+$ cells) isolated from the spleen of a chronically infected mouse 14 dpi. **c** Example of flow cytometry plots showing PD-1 and *Nr4a1*-GFP expression in P14 cells isolated from various tissues at the indicated time-points into chronic infection. **d** Bar plots show mean ± SD of the frequency or medians of GFP$^+$ P14-*Nr4a1*-GFP cells isolated at the indicated time points after infection. Each dot represents an individual mouse. One of two experiments is shown (n=3-4 mice). Source data are provided as a Source Data File.

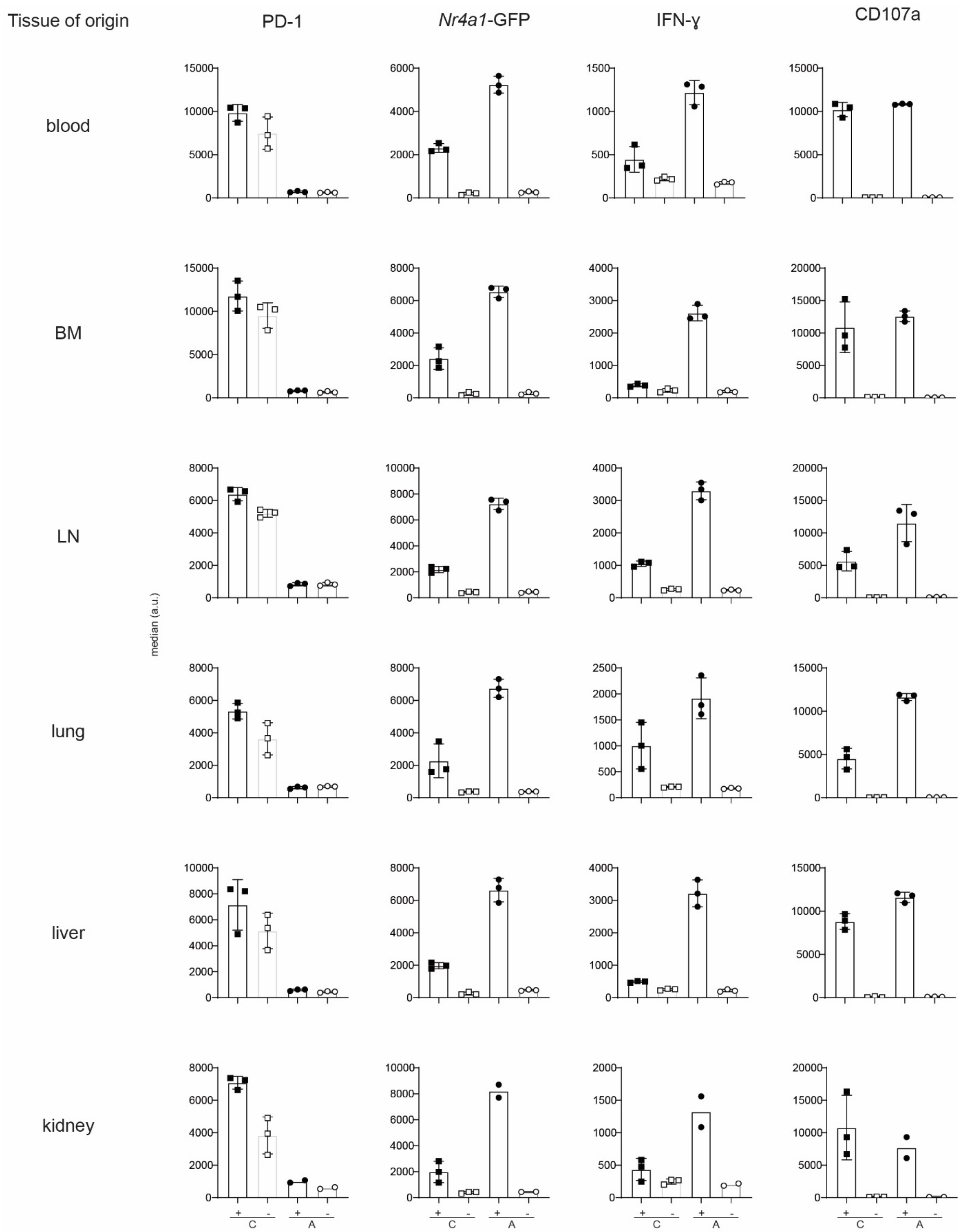


**Supplementary Fig. 2: *In vitro* restimulation of sorted P14 cells harvested from different organs.** CD45.1$^+$ P14-*Nr4a1*-GFP cells were adoptively transferred into CD45.2$^+$ mice one day prior infection with a high ($2x10^6$ ffu for chronic (C) infection) or low (200 ffu to induce an acute (A) infection) dose of LCMV clone 13. Three weeks into chronic infection, P14 cells were sorted from different tissues (lymph nodes (LN,) bone marrow (BM), lungs, liver, blood, and kidney) and stimulated in vitro for six hours with plate-bound antibodies. Various

markers were quantified (average of medians ± SD are depicted) in cells that responded ($CD107a^{+}$), denoted by plus signs, and cells that did not ($CD107a^{-}$), denoted by minus signs. One of two experiments is shown (n=3 mice). Each dot represents an individual mouse. Source data are provided as a Source Data File.

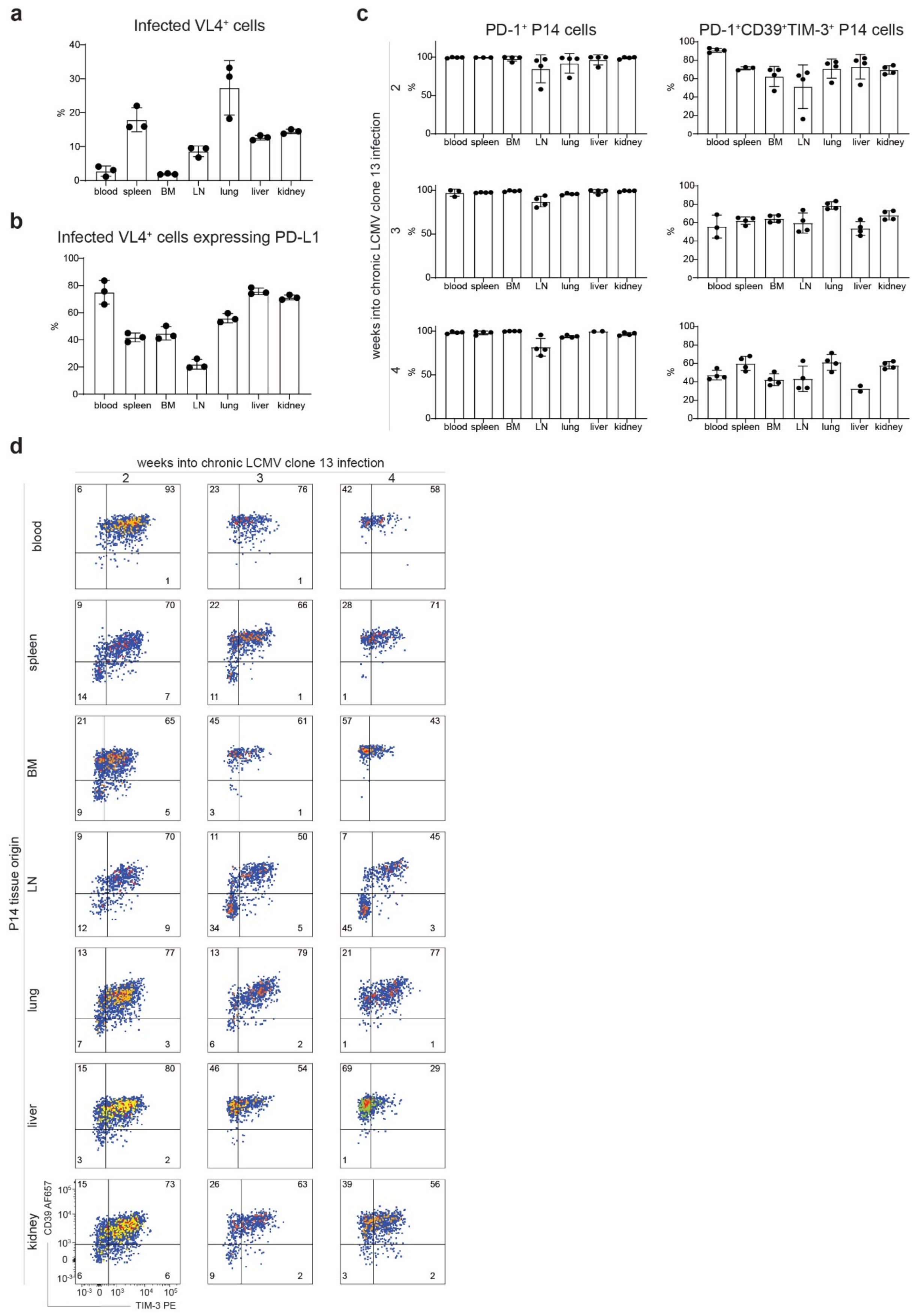


**Supplementary Fig. 3: PD-L1 expression on infected cells and inhibitory receptor expression on P14 cells.**

Percentage of infected cells (gated on alive cells (**a**) or gated on alive VL4⁺ cells (**b**) expressing PD-L1 are

shown for seven tissues harvested from chronically LCMV infected mice 21 days into chronic infection (n = 3 mice). **c-d** P14-*Nr4a1*-GFP cells were adoptively transferred into hosts one day prior high dose LCMV infection. Graphs show the frequency of PD-$1^+$ or PD-$1^+$CD$39^+$TIM-$3^+$ of P14 cells isolated from various tissues at different time points after infection (**c**). Example of flow cytometry plots show TIM-3 and CD39 expression in P14 cells (gated on alive single CD$8^+$ CD$45.1^+$ cells) isolated from different tissues at indicated time-points into chronic infection (**d**). Bar plots show mean ± SD. Each dot represents an individual mouse. One of two experiments is shown (n=2-4 mice). Source data are provided as a Source Data File.

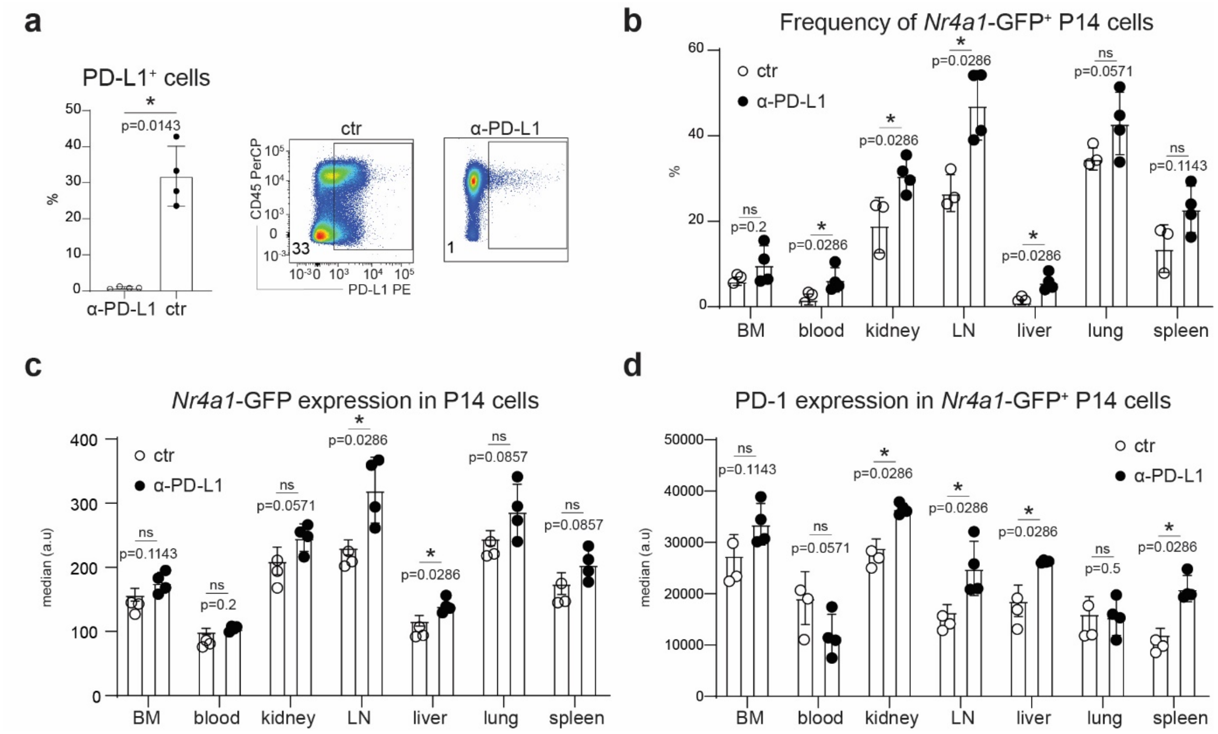


**Supplementary Fig. 4: PD-L1 blockade increases TCR signaling in various tissues.** CD45.1$^+$ P14-*Nr4a1*-GFP were adoptively transferred into naïve CD45.2$^+$ hosts one day prior high dose LCMV clone 13 infection. 21 days into chronic infection, one group received one dose of 200 µg α-PD-L1 blocking antibody intravenously. The mice were sacrificed six hours after treatment. **a** Representative flow cytometry plots (cells isolated from the spleen, gated on alive single cells). PD-L1 was stained to check blockade efficiency with the same clone used for blocking. Fraction of *Nr4a1*-GFP$^+$ cells (gated on single alive CD8$^+$ CD45.1$^+$ P14 cells) (**b**), mean of GFP (**c**) and PD-1 (**d**) (gated on single alive CD8$^+$ CD45.1$^+$ P14 GFP$^+$ cells) are shown for cells isolated from various tissues. Bar plots represent mean ± SD. * denotes a significant p-value ≤ 0.05 and ns non-significance (one-tailed Mann-Whitney test without correction for multiple comparisons). One of three experiments is shown (n=4 mice). Each dot represents an individual mouse. Source data are provided as a Source Data File.

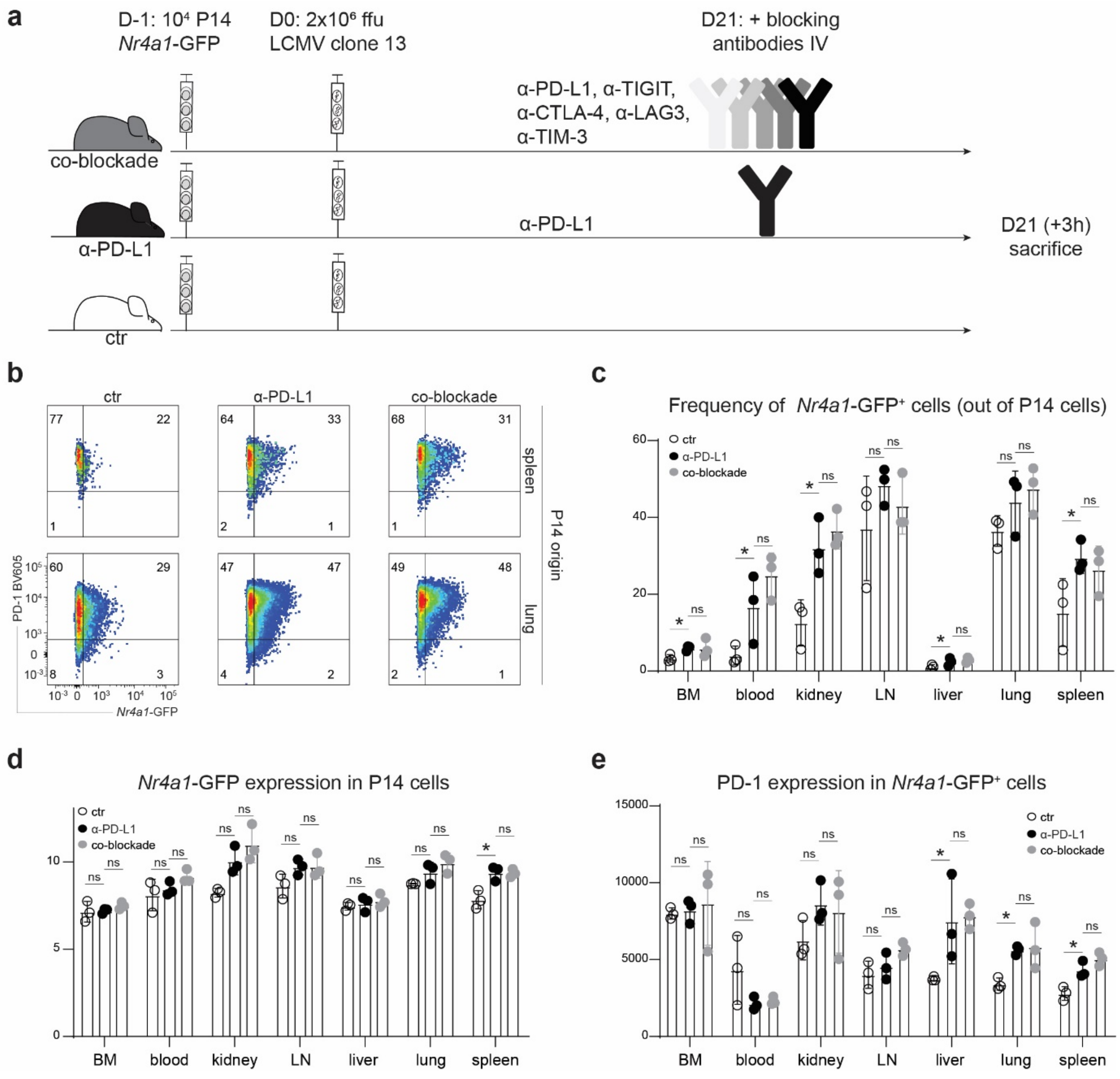


**Supplementary Fig. 5: Short-term inhibitory receptors co-blockade does not increase TCR signaling compared to PD-L1 blockade alone. a** CD45.1$^+$ P14-*Nr4a1*-GFP were adoptively transferred into naïve CD45.2$^+$ hosts one day prior high dose LCMV clone 13 infection. 21 days post-infection, mice received one dose of α-PD-L1, α-TIGIT, α-CTLA-4, α-LAG3, α-TIM-3 (co-blockade), one dose of α-PD-L1 blocking antibody alone intravenously or no antibody (ctr). The mice were sacrificed 3 hours after treatment. **b** Representative flow cytometry plots showing *Nr4a1*-GFP and PD-1 signal in the three experimental groups, control (ctr) and treated (α-PD-L1 and co-blockade) in P14 cells (gated on alive single CD8$^+$ CD45.1$^+$ cells) isolated from spleen or lung. Frequency of *Nr4a1*-GFP$^+$ cells (out of alive single CD8$^+$ CD45.1$^+$ cells) (**c**), median of GFP (**d**) and PD-1 (**e**) (gated on alive single CD8$^+$ CD45.1$^+$ GFP$^+$ cells) are shown for cells isolated from different tissues. Bar plots represent mean ± SD. * denotes p-value = 0.05 and ns non-significance (Mann-Whitney one tailed-test without correction for multiple comparisons). One of two experiments is shown (n=3 mice). Each dot represents an individual mouse. Source data are provided as a Source Data File.

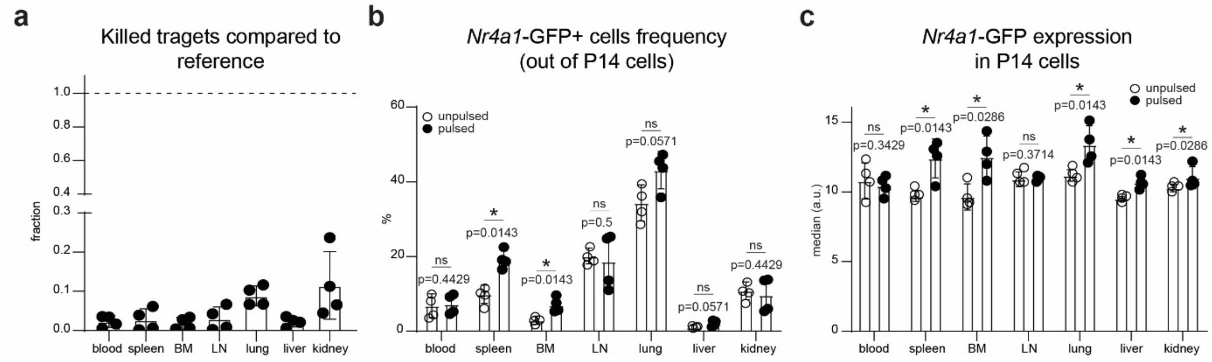


**Supplementary Fig. 6: *In vivo* killing assay three weeks into chronic infection.** CD45.1$^{+}$ P14-*Nr4a1*-GFP cells were adoptively transferred into naïve CD45.2$^{+}$ mice one day prior high dose infection. 21 days into chronic infection, the hosts received $GP_{33\text{-}41}$-pulsed or unpulsed targets together with a reference population. Three hours later the mice were sacrificed. **a** Fraction of recovered targets (calculated based on the reference population) in different tissues isolated from unpulsed or pulsed groups is shown. Frequency of GFP$^{+}$ cells (gated on alive single CD8$^{+}$ CD45.1$^{+}$ P14 cells) (**b**) and medians of GFP (gated on alive single CD8$^{+}$ CD45.1$^{+}$ GFP$^{+}$ cells) (**c**) are shown. Bar plots represent mean ± SD. * denotes a significant p-value ≤ 0.05 and ns non-significance (Mann-Whitney one tailed-test without correction for multiple comparisons). Each dot represents an individual mouse. One of three experiments is shown (n=4 mice). Source data are provided as a Source Data File.

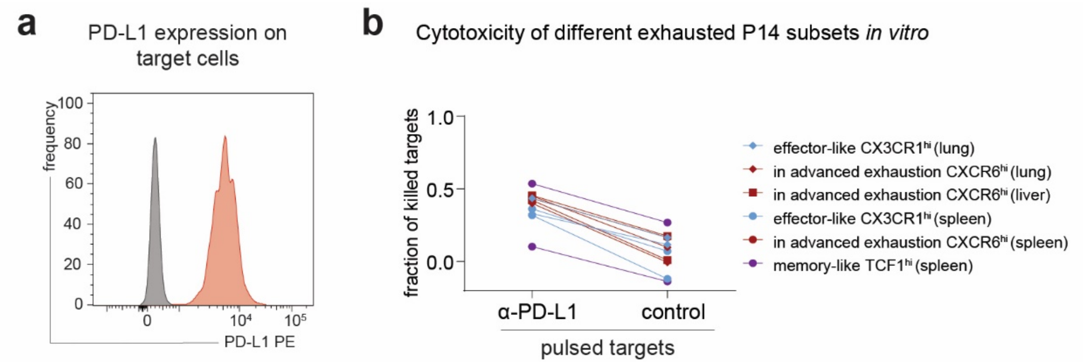


**Supplementary Fig. 7**: ***In vitro* killing assay three weeks into chronic infection.** CD45.1$^+$ P14-*Tcf7* cells were adoptively transferred into naïve CD45.2$^+$ mice one day prior high dose infection. P14 cells were sorted from chronically infected mice 21 dpi based on CX3CR1, CXCR6, and TCF1-GFP expression into advanced exhausted CXCR6$^{hi}$CX3CR1$^{neg}$, effector-like CX3CR1$^{hi}$CXCR6$^{lo}$, and memory-like GFP$^{hi}$ cells. Sorted populations were incubated with GP$_{33\text{-}41}$ pulsed EL4 target cells at 5:1 E/T ratio. **a** EL4 cells were incubated with IFN-γ at 100 U/mL for 6 hours prior pulsing to induce PD-L1 upregulation (red histogram). For one condition, PD-L1 expression was blocked with 30 µg/mL α-PD-L1 for one hour at 37ºC (gray histogram). **b** The graph shows fraction of killed targets; lines denote paired samples (same P14 sorted population incubated with pulsed targets expressing PD-L1 (control) or pulsed targets in presence of blocking PD-L1 antibody (α-PD-L1)). One of two experiments is shown. Each data point represents a sorted population from 2 or 3 pooled mice. n=2 groups of pooled mice. 2 and 3 mice were pooled per group to increase recovered cell numbers. Source data are provided as a Source Data File.

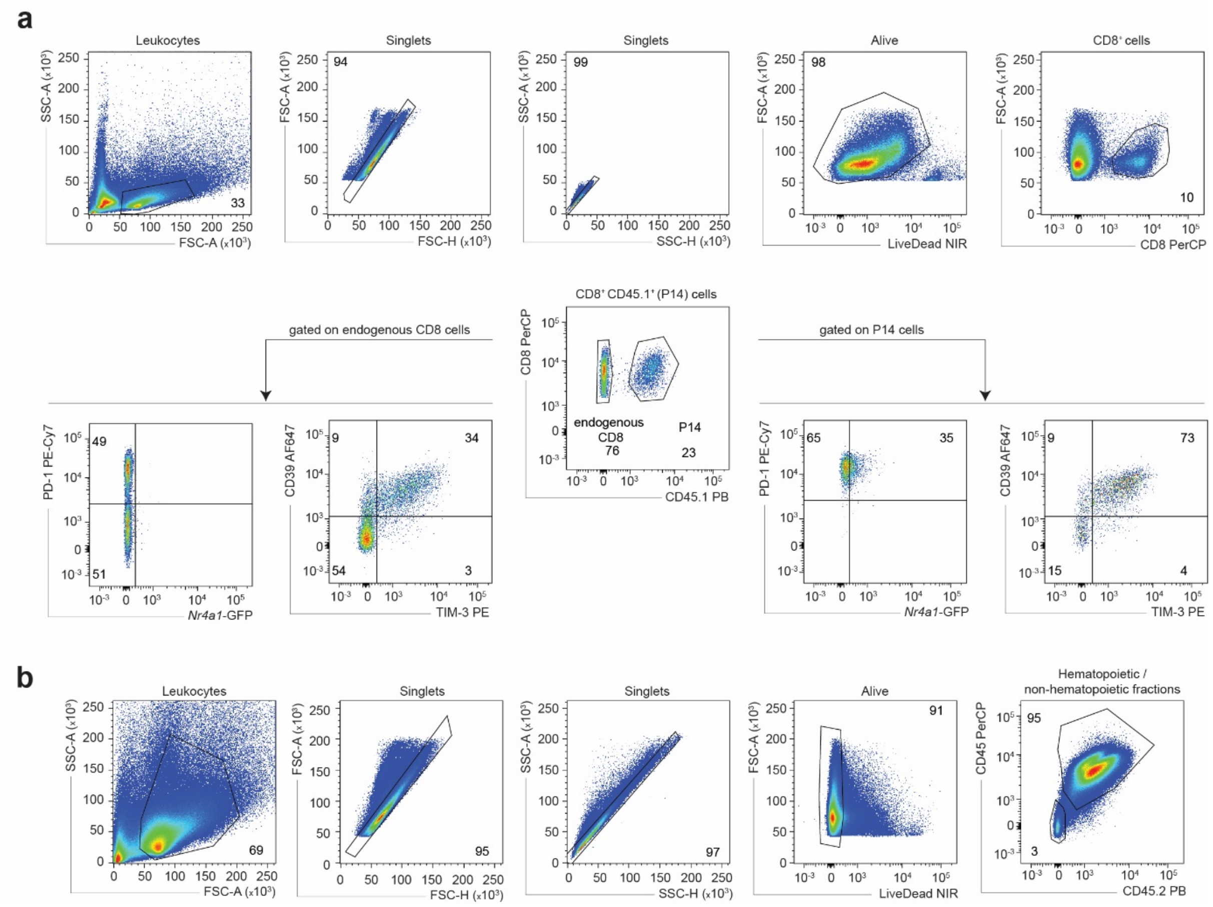


**Supplementary Fig. 8: Flow cytometry gating strategy. a** For all samples the following gating strategy was used: leukocytes (SSC-A/FSC-A), exclusion of doublets (FSC-H/FSC-A and SSC-H/SSC-A), alive cells (Near IR/FSC-A). For P14 staining, cells were gated on CD8 (FSC-A/CD8 gate), then CD45.1 (CD8/CD45.1 gate). *Nr4a1*-GFP gates were set using endogenous CD8 cells (gated on alive single CD8+ cells) not expressing the transgene from the same mouse. Inhibitory receptor gates (PD-1, CD39, TIM-3) were set on endogenous CD8 cell population where distinct populations could be clearly identified. **b** For hematopoietic or non-hematopoietic populations, a wider FSC-A/SSC-A gate was used (including all cells). In order to distinguish the hematopoietic from the non-hematopoietic fraction, CD45 and CD45.2 staining was used.
