# Supplementary material for: Exhausted CD8+ T cells exhibit low and strongly inhibited TCR signaling during chronic LCMV infection
Source: Nat Commun. 2020 Sep 8;11:4454. doi: 10.1038/s41467-020-18256-4 (PMC7479152; doi:10.1038/s41467-020-18256-4)
Supplement: Supplementary file 3 — Reporting Summary [file 41467_2020_18256_MOESM3_ESM.pdf]

nature research

Corresponding author(s): Annette Oxenius

Last updated by author(s): Jul 27, 2020

Reporting Summary

Nature Research wishes to improve the reproducibility of the work that we publish. This form provides structure for consistency and transparency in reporting. For further information on Nature Research policies, see our Editorial Policies and the Editorial Policy Checklist.

Statistics

For all statistical analyses, confirm that the following items are present in the figure legend, table legend, main text, or Methods section.

n/a	Confirmed	
☐	☒	The exact sample size (n) for each experimental group/condition, given as a discrete number and unit of measurement
☐	☒	A statement on whether measurements were taken from distinct samples or whether the same sample was measured repeatedly
☐	☒	The statistical test(s) used AND whether they are one- or two-sided<br>*Only common tests should be described solely by name; describe more complex techniques in the Methods section.*
☒	☐	A description of all covariates tested
☒	☐	A description of any assumptions or corrections, such as tests of normality and adjustment for multiple comparisons
☐	☒	A full description of the statistical parameters including central tendency (e.g. means) or other basic estimates (e.g. regression coefficient) AND variation (e.g. standard deviation) or associated estimates of uncertainty (e.g. confidence intervals)
☐	☒	For null hypothesis testing, the test statistic (e.g. F, t, r) with confidence intervals, effect sizes, degrees of freedom and P value noted<br>*Give P values as exact values whenever suitable.*
☒	☐	For Bayesian analysis, information on the choice of priors and Markov chain Monte Carlo settings
☒	☐	For hierarchical and complex designs, identification of the appropriate level for tests and full reporting of outcomes
☒	☐	Estimates of effect sizes (e.g. Cohen's d, Pearson's r), indicating how they were calculated

Our web collection on statistics for biologists contains articles on many of the points above.

Software and code

Policy information about availability of computer code

Data collection	Data were collected using BD FACSDiva (v8.0.1) and LSRII, Canto or Fortessa cytometers. Sequencing data was generated on Illumina platform.
Data analysis	Flow cytometry data were analyzed in Flowjo (versions 10.6.1 and 9). Data was exported into Graphpad prism (version 8.2.0) for statistical analysis. Sequencing data were mapped with STAR aligner and analyzed with DESeq2 in R (3.6.0). Custom scripts are available here: https://gitlab.com/claassen_lab/tcrsignaling

For manuscripts utilizing custom algorithms or software that are central to the research but not yet described in published literature, software must be made available to editors and reviewers. We strongly encourage code deposition in a community repository (e.g. GitHub). See the Nature Research guidelines for submitting code & software for further information.

Data

Policy information about availability of data

All manuscripts must include a data availability statement. This statement should provide the following information, where applicable:

- Accession codes, unique identifiers, or web links for publicly available datasets
- A list of figures that have associated raw data
- A description of any restrictions on data availability

The RNAseq raw data files can be found at the European Nucleotide Archive under accession number #PRJEB38896 (https://www.ebi.ac.uk/ena/browser/view/PRJEB38896). The flow cytometry files supporting the findings are available from the corresponding author upon request. The source data underlying F1a, 2b-d, 3, 4c, 5c, d, 6c–e, 7 c, e-g, Supplementary Figs. 1a, d, 2, 3a-c, 4b–d, 5c–e, 6a-c, 7b are provided in the Source Data File.

Field-specific reporting

Please select the one below that is the best fit for your research. If you are not sure, read the appropriate sections before making your selection.

☒ Life sciences ☐ Behavioural & social sciences ☐ Ecological, evolutionary & environmental sciences

For a reference copy of the document with all sections, see nature.com/documents/nr-reporting-summary-flat.pdf

Life sciences study design

All studies must disclose on these points even when the disclosure is negative.

Sample size

Between 3-4 mice were used per group within each experiment. This number was based on previous experience with the infection model (Agnellini et al. (2007), Blackburn et al. (2008)), the availability of the mice, and feasibility of the experiment.

Data exclusions

Samples with fewer than 50 cells of interest (in this case transgenic P14 cells) were excluded because this threshold for analysis was pre-established. This was the case for 2 samples (out of 4) isolated from the liver 4 wpi in Supplementary Figure 1d and Supplementary Figure 3c and a sample isolated from kidney 3 weeks post acute infection in Supplementary Figure 2.

Replication

Experiments (except the RNAseq experiment shown in Fig. 1) were performed at least twice with similar conditions and considered reproducible if results were comparable.

Randomization

Mice were randomly distributed amongst groups at the start of each experiment.

Blinding

Blinding was not performed, due to technical reasons. For in vivo experiments it is required by local authorities to write on the cage cards all procedures the mice are subjected to.

Reporting for specific materials, systems and methods

We require information from authors about some types of materials, experimental systems and methods used in many studies. Here, indicate whether each material, system or method listed is relevant to your study. If you are not sure if a list item applies to your research, read the appropriate section before selecting a response.

Materials & experimental systems

n/a	Involved in the study	
☐	☒	Antibodies
☐	☒	Eukaryotic cell lines
☒	☐	Palaeontology and archaeology
☐	☒	Animals and other organisms
☒	☐	Human research participants
☒	☐	Clinical data
☒	☐	Dual use research of concern

Methods

n/a	Involved in the study	
☒	☐	ChIP-seq
☐	☒	Flow cytometry
☒	☐	MRI-based neuroimaging

Antibodies

Antibodies used

Marker Fluorophore Supplier Catalog number Clone Purpose Dilution Reactivity
B220 Biotin BioLegend 103204 RA3-6B2 Enrichment 1/100 Mouse
CD107a PE BioLegend 121612 1D4B Flow 1/1000 Mouse
CD39 AF647 BioLegend 143807 Duha59 Flow 1/200 Mouse
CD4 biotin BioLegend 100404 GK1.5 Flow 1/200 Mouse
CD44 BV510 BioLegend 102116 IM7 Flow 1/200 Mouse
CD45 PerCP BD Biosciences 557235 30-F11 Flow 1/200 Mouse
CD45.1 APC BioLegend 110714 A20 Flow 1/200 Mouse
CD45.1 BV421 BioLegend 110731 A20 Flow 1/200 Mouse
CD45.1 PB BioLegend 110722 A20 Flow 1/200 Mouse
CD45.1 PB BioLegend 110722 A20 Flow 1/200 Mouse
CD45.1 PE BioLegend 110708 A20 Flow 1/200 Mouse
CD45.1 BV711 BioLegend 110739 A20 Flow 1/200 Mouse
CD45.2 Percp BioLegend 109825 104 Flow 1/200 Mouse
CD45.2 BV421 BioLegend 109831 104 Flow 1/200 Mouse
CD8 Percp BioLegend 100732 53-6.7 Flow 1/200 Mouse
CD8 BV605 BioLegend 100744 53-6.7 Flow 1/200 Mouse
CD8 BUV395 BioLegend 102116 53-6.7 Flow 1/200 Mouse
CD8 BV711 BioLegend 100714 53-6.7 Flow 1/200 Mouse
CTLA-4 - BioXcell BE0032 UC10-4F10-11 in vivo blocking 200 ug/mouse Mouse

CX3CR1 PerCPCy5.5 Biolegend 149010 SA011F11 Flow 1/200 Mouse
CXCR6 PE Biolegend 151104 SA051D1 Flow 1/200 Mouse
IFNg PB BioLegend 505818 XMG1.2 Flow 1/200 Mouse
LAG-3 - BioXcell BE0174 C9B7W in vivo blocking 200 ug/mouse Mouse
PD-1 PECy7 BioLegend 135215 29F.1A12 Flow 1/200 Mouse
PD-1 BV605 BioLegend 135219 29F.1A12 Flow 1/200 Mouse
PD-L1 BV421 BioLegend 124315 10F.9G2 Flow 1/200 Mouse
PD-L1 PE BioLegend 124308 10F.9G2 Flow 1/200 Mouse
PD-L1 - BioXcell BE00101 10F.9G2 in vivo blocking 200 ug/mouse Mouse
TIGIT - provided by Dr. Nicole Joller (UZH) - 1B4 in vivo blocking 200 ug/mouse Mouse
Tim-3 PE BioLegend 119703 RMT3-23 Flow 1/200 Mouse
TIM-3 - BioXcell BE0115 RMT3-23 in vivo blocking 200 ug/mouse Mouse
VL4 FITC Generated in-house - - Flow/Focus Forming Assay 1/50 Mouse
CD28 - Biolegend 102112 37.51 in vitro restimulation 1 ug/mL Mouse
CD3 - Biolegend 100340 145-2C11 in vitro restimulation 1 ug/mL Mouse

Validation

The table below contains the validation performed by the manufacturer:
Supplier Catalog number Clone Validation manufacturer
BioLegend 103204 RA3-6B2 Staining on C57BL/6 mouse splenocytes
BioLegend 121612 1D4B Thioglycollate-elicited BALB/c mouse peritoneal macrophages
BioLegend 143807 Duha59 Staining on C57BL/6 mouse splenocytes
BioLegend 100404 GK1.5 Staining on C57BL/6 mouse splenocytes
BioLegend 102116 IM7 Staining on C57BL/6 mouse splenocytes
BD Biosciences 557235 30-F11 This antibody has been tested by immunofluorescent staining by flow cytometric analysis.
BioLegend 110714 A20 Staining on C57BL/6 mouse splenocytes
BioLegend 110731 A20 Staining on C57BL/6 mouse splenocytes
BioLegend 110722 A20 Staining on C57BL/6 mouse splenocytes
BioLegend 110722 A20 Staining on C57BL/6 mouse splenocytes
BioLegend 110708 A20 Staining on C57BL/6 mouse splenocytes
BioLegend 110739 A20 Staining on C57BL/6 mouse splenocytes
BioLegend 109825 104 Staining on C57BL/6 mouse splenocytes
BioLegend 109831 104 Staining on C57BL/6 mouse splenocytes
BioLegend 100732 53-6.7 Staining on C57BL/6 mouse splenocytes
BioLegend 100744 53-6.7 Staining on C57BL/6 mouse splenocytes
BioLegend 102116 53-6.7 Staining on C57BL/6 mouse splenocytes
BioLegend 100714 53-6.7 Staining on C57BL/6 mouse splenocytes
BioXcell BE0032 UC10-4F10-11 Western Blot staining of purified CTLA-4
Biolegend 149010 SA011F11 Staining on C57BL/6 mouse splenocytes
Biolegend 151104 SA051D1 Staining on C57BL/6 mouse splenocytes
BioLegend 505818 XMG1.2 PMA and Ionomycin-stimulated (6hrs) BALB/c splenocytes
BioXcell BE0174 C9B7W Western Blot staining of purified LAG-3
BioLegend 135215 29F.1A12 Con-A stimulated C57BL/6 mouse splenocytes
BioLegend 135219 29F.1A12 Con-A stimulated C57BL/6 splenocytes
BioLegend 124315 10F.9G2 Staining on C57BL/6 mouse splenocytes
BioLegend 124308 10F.9G2 Staining on C57BL/6 mouse splenocytes
BioXcell BE00101 10F.9G2 Western Blot staining of purified PD-L1
provided by Dr. Nicole Joller (UZH) - 1B4 Dixon et al. (2018)
BioLegend 119703 RMT3-23 Mouse TIM-3 transfected cells stained with anti-mouse CD366
BioXcell BE0115 RMT3-23 Western Blot staining of purified TIM-3
Generated in-house - - Staining of splenocytes from a chronically infected mouse with LCMV and splenocytes from a naïve (uninfected) mouse
Biolegend 102112 37.51 Staining on C57BL/6 mouse splenocytes
Biolegend 100340 145-2C11 Staining on C57BL/6 mouse splenocytes

Eukaryotic cell lines

Policy information about cell lines

Cell line source(s)

BHK21 [C13] (ATCC® CCL10), MC57G (ATCC® CRL-2295), VL-4 hybridoma Cat#:006A-EVA92, EL4 (ATCC® TIB-39™)

Authentication

The cell lines were not authenticated in our lab.

Mycoplasma contamination

Cell lines used for virus and antibody production (BHK-21 and VL-4 hybridoma) were free of Mycoplasma contamination. MC57G and EL4 cells tested positive for mycoplasma.

Commonly misidentified lines
(See ICLAC register)

No commonly misidentified cell lines were used in the study.

Animals and other organisms

Policy information about studies involving animals; ARRIVE guidelines recommended for reporting animal research

Laboratory animals	Wild-type CC57BL/6J males purchased from Janvier Elevage, Nr4a1-GFP mice expressing a GFP under the control of the NUR77 promoter, Tcf7-GFP mice expressing GFP under the control of Tcf7 (encoding TCF1) promoter, P14 transgenic (CD45.1) mice expressing a TCR specific for LCMV peptide GP33–41 were housed at 24C and 50% humidity and bred under specific pathogen–free conditions. Mice were exposed to a 12:12h light-dark cycle with unrestricted access to water and food. Mice were between 6-16 weeks of age at the start of each experiment.
Wild animals	No wild animals were used in the study.
Field-collected samples	No field collected samples were used in the study.
Ethics oversight	This study was conducted in accordance to the guidelines of the animal experimentation law (SR 455.163; TVV) of the Swiss Federal Government. The protocol was approved by Cantonal Veterinary Office of the canton Zurich, Switzerland (experimental permits 147/2014, 115/2017).

Note that full information on the approval of the study protocol must also be provided in the manuscript.

Flow Cytometry

Plots

Confirm that:

☒ The axis labels state the marker and fluorochrome used (e.g. CD4-FITC).

☒ The axis scales are clearly visible. Include numbers along axes only for bottom left plot of group (a 'group' is an analysis of identical markers).

☒ All plots are contour plots with outliers or pseudocolor plots.

☒ A numerical value for number of cells or percentage (with statistics) is provided.

Methodology

Sample preparation	Mice were sacrificed with carbon dioxide. Blood was taken from the heart or vena cava. The mice were then perfused with 20 mL PBS, and organs (spleen, bone marrow, lymph nodes, lung, liver, and kidney) were isolated. Lungs, liver, and kidney were cut into pieces and digested in complete RPMI (Bioconcept) (RPMI-1640 containing 10% fetal bovine serum (Omnilab), 2 mM L-glutamine (Gibco), 1% penicillin-streptomycin (Gibco), 1 mM sodium pyruvate (Gibco), 50 nM betamercapthoethanol, 0.1 mM non-essential (glycine, L-alanine, L-asparagine, L-aspartic acid, L-glutamic acid, L-proline, L-serine) amino acids (Gibco), 20 mM HEPES (Gibco)) supplemented with 2.4 mg/ml collagenase type I (Gibco), and 0.2 mg/ml DNase I (Roche Diagnostics, Rotkreuz, Switzerland) for 30 minutes at 37C. Spleens and lymph nodes were mashed through 70 µm filter with a syringe (1 mL) plunger. Cell suspensions were filtered (70 µm) and treated with ammonium-chloride-potassium buffer (150 mM NH_4Cl, 10 nM $KHCO_3$, 0.1 mM EDTA in water) to lyse erythrocytes for 5 min at room temperature. Cell suspension from lungs, liver, and kidney were enriched for lymphocytes by centrifugation over Percoll density centrifugation (30% (v/v) Percoll in PBS) at 4C (500 g) for 30 minutes.
Instrument	Multi-parametric flow cytometric data was acquired using LSRII, Canto or Fortessa flow cytometers (BD Biosciences).
Software	Flow cytometry data were analyzed in Flowjo (versions 10.6.1 and 9). Data was exported into Graphpad prism (version 8.2.0). Sequencing data were analyzed in R with limma, DEseq2, ggplot2 packages.
Cell population abundance	Purity of the sorted P14 cells used for RNAseq was above 95% (as determined by flow cytometry post-sort).
Gating strategy	For all samples the following gating strategy was used: leukocytes (SSC-A/FSC-A), exclusion of doublets (FSC-H/FSC-A and SSC-H/SSC-A), alive cells (Near IR/FSC-A).<br>For P14 staining, cells were gated on CD8 (FSC-A/CD8 gate), then CD45.1 (CD8/CD45.1 gate).<br>For hematopoietic or non-hematopoietic populations, a wider FSC-A/SSC-A gate was used (including all cells). TO distinguish the hematopoietic from the non-hematopoietic fraction, CD45 and CD45.2 staining was used.<br>Nr4a1-GFP gates were set using endogenous CD8 cells (gated on alive single CD8+ cells) not expressing the transgene from the same mouse. Inhibitory receptor gates (PD-1, CD39, TIM-3) were set on endogenous CD8 cell population where distinct populations could be clearly identified.<br>VL4-FITC gates were determined by using a control (uninfected) mouse.

☒ Tick this box to confirm that a figure exemplifying the gating strategy is provided in the Supplementary Information.
