# Supplementary material for: Exhausted CD8+ T cells exhibit low and strongly inhibited TCR signaling during chronic LCMV infection
Source: Nat Commun. 2020 Sep 8;11:4454. doi: 10.1038/s41467-020-18256-4 (PMC7479152; doi:10.1038/s41467-020-18256-4)
Supplement: Supplementary file 4 — Source Data [file 41467_2020_18256_MOESM4_ESM.zip › 243187_2_supp_4795034_q7497c.pdf]

Figure 1a

	acute_unstimulated			chronic_unstimulated			chronic_stimulated			acute_stimulated		
Gene	1	2	3	1	2	3	1	2	3	1	2	3
Tppp3	0.10446	1.04632	1.00855	-0.0235	-0.9751	-0.684	-1.2499	-1.2067	-1.09399	0.7793	1.18458	1.11
Ifitm1	0.41204	0.92612	1.0863	-1.073	-0.7361	-1.03303	-0.8018	-1.0203	-0.96294	1.0421	1.10025	1.0604
Gzmm	0.70822	1.04767	1.0417	-0.5613	-1.2994	-1.02741	-0.8504	-0.9724	-0.92962	0.9983	0.80855	1.0361
Irak3	0.83158	1.14319	0.66039	-1.3887	-0.9439	-0.35857	-1.099	-0.9311	-0.82338	0.8019	1.03989	1.0678
H2-Q10	0.78789	0.9657	1.0815	-1.0025	-0.8954	-0.40478	-0.7961	-1.1329	-1.36017	0.9513	0.8564	0.949
Itgax	1.11875	0.96505	0.79562	-0.9599	-0.7305	-0.22184	-1.4421	-1.0668	-1.07295	1.0125	0.8373	0.7647
Il12rb2	1.04062	0.98545	0.78924	-0.7505	-0.8049	-0.56836	-1.3521	-0.8684	-1.2593	0.794	1.03052	0.9637
Fcgrt	1.26616	1.02749	1.16504	-0.8815	-0.5366	-1.11871	-0.715	-1.2269	-1.07913	0.7835	0.71679	0.5989
P2ry12	1.18165	1.46619	0.95573	-0.8274	-0.5384	-0.95796	-1.2579	-0.8347	-1.05817	0.6701	0.80024	0.4006
Wfikkn2	0.99156	1.35222	0.76923	-1.1231	-1.0235	-0.7325	-1.1105	-1.0226	-0.52145	1.2068	0.54545	0.6683
Slco3a1	1.22633	1.00222	0.85504	-0.8564	-0.4912	-0.87582	-1.1265	-1.5894	-0.54205	0.8616	0.70602	0.8301
Klf3	1.55376	1.00552	1.51739	-0.3446	0.14064	0.14627	-1.1948	-1.1471	-1.36634	0.4516	-0.3203	-0.4421
S1pr5	1.37405	1.36465	1.27068	-0.7344	-0.7283	-0.02458	-0.8632	-1.4132	-1.21467	0.1913	0.45228	0.3254
Med12l	1.20687	1.00005	1.22364	-0.4588	-1.2687	0.05969	-1.0635	-1.3776	-1.05208	0.4483	0.81993	0.4622
As3mt	1.45545	1.19984	1.14292	-0.3604	-1.2493	-0.96396	-0.9554	-0.851	-0.9733	0.6738	0.22942	0.6519
Klrg1	0.97612	1.55015	1.44265	-0.6067	-0.7975	-0.81099	-0.9496	-1.1938	-0.96943	0.5534	0.36179	0.444
Ccr2	1.24373	1.52146	1.36953	-0.3144	-0.5009	-0.71994	-1.17	-0.9993	-1.31382	0.3626	0.13037	0.3906
Acss2	1.47156	1.33895	1.02236	-1.1537	-0.5377	-0.28592	-1.2731	-0.6865	-1.15279	0.8817	0.07423	0.301
Esm1	1.64238	1.09866	1.08598	-0.336	-1.0186	-0.32038	-1.2107	-0.9824	-1.19079	0.8045	0.01917	0.4082
Kcnj8	1.37819	1.14033	1.47342	-0.3636	-0.8867	0.0925	-1.1851	-1.1637	-1.10708	0.4871	-0.4181	0.5529
Kit	-1.36452	-0.84378	-1.3708	1.21887	1.72459	1.093	-0.3731	0.46134	-0.56249	-0.301	0.36243	-0.0443
Osgin1	-0.36529	-0.58562	-0.8046	1.54376	1.53128	1.47258	-0.7376	0.2379	0.3198	-0.885	-0.8948	-0.8325
Cyth3	-0.3173	-0.83286	-1.2085	1.3346	1.4268	1.47441	0.04559	0.5453	0.20535	-0.848	-1.0304	-0.7953
Ltf	-1.01743	-1.12679	-1.0942	0.94822	0.78519	1.49901	1.00663	0.21884	0.90496	-0.929	-0.2252	-0.9705
Ccr9	-1.03886	-0.76695	-0.7798	0.96216	1.32133	1.03618	0.79589	0.69668	0.79005	-0.929	-1.3551	-0.7323
2900026A02Rik	-1.03249	-0.92194	-0.7639	1.04755	1.26299	1.11011	1.04548	0.61784	0.53734	-0.984	-0.9952	-0.9234
Cd7	-1.03593	-1.03617	-1.0304	1.12926	1.19242	0.95945	0.97915	0.59774	0.81099	-0.823	-0.8341	-0.9096
Adgrg1	-0.85236	-1.42755	-0.7769	1.00092	1.21764	1.28161	0.60477	0.83321	0.60721	-0.754	-0.9085	-0.8262
Cd200r1	-0.88092	-0.96957	-0.9115	1.43288	1.39229	1.09737	0.49745	0.46323	0.57456	-0.752	-0.9	-1.0439
Tmprss6	-0.95734	-0.96382	-0.9354	1.38738	1.25322	1.31969	0.30945	0.73139	0.42145	-0.932	-0.9407	-0.6931
Crmp1	-0.88853	-0.4138	-0.2813	1.03115	0.69854	1.24879	0.67655	0.99471	0.64492	-1.625	-1.334	-0.7517
St14	-0.8771	-0.44068	-0.5302	-0.0221	1.05141	0.91102	0.99675	1.41399	0.9178	-1.247	-0.9047	-1.2688
Tnfsf4	-1.03724	-0.78903	-0.8346	0.75434	0.82181	1.03607	1.10991	0.89763	1.05963	-1.153	-1.1017	-0.7637
Cd38	-0.56709	-0.90621	-0.8067	0.74073	0.82428	0.92125	1.09971	1.04853	0.97973	-1.424	-0.889	-1.0212
1700019D03Rik	-0.91355	-0.91211	-0.9533	0.19577	0.86305	0.84458	1.27779	1.20455	1.14688	-0.93	-0.9392	-0.8849
Epdr1	-0.85252	-0.84709	-0.986	0.71654	1.08257	0.29781	1.24036	1.15923	1.0738	-0.962	-0.951	-0.9719
Slc16a11	-0.92774	-0.9396	-0.9365	0.62415	0.79122	0.58243	1.46149	1.10683	1.02796	-0.971	-0.978	-0.8416
Vax2	-0.96129	-0.53274	-1.0847	0.52425	1.29631	1.2398	0.65837	0.95867	0.88525	-0.899	-1.0941	-0.9911
Tox2	-0.92583	-1.10614	-1.1125	0.80841	0.91351	0.90805	1.02355	1.00658	1.04689	-0.732	-0.8953	-0.9356
Serpina3g	-1.1534	-1.01844	-0.9296	0.84251	1.07317	0.72683	1.02458	1.12509	0.88327	-1.07	-0.8114	-0.693
Klrb1b	-1.13728	-0.86535	-0.9393	0.95659	1.25837	0.58733	0.86195	1.12755	0.85667	-0.737	-1.0072	-0.9624
Cxcl10	-1.28672	-1.51889	-1.2811	0.89908	1.07698	1.08727	0.67222	0.78854	0.67919	-0.777	-0.327	-0.0127
Pdcd1	-1.64572	-1.51217	-1.4653	0.72561	0.51763	0.59459	0.88151	1.02435	0.89903	-0.211	0.01817	0.1737
Cd83	-1.73144	-1.21943	-1.3748	0.83201	0.7828	0.9064	0.87722	0.86208	0.72115	-0.659	0.12746	-0.1247
Myo1e	-1.12887	-1.34912	-1.0063	0.36508	0.14518	0.61677	0.91613	1.67414	1.28196	-0.71	-0.0528	-0.7517
Lcn2	-1.05627	-0.9301	-1.1191	0.44352	0.21523	1.02515	1.36602	0.84094	1.44882	-0.942	-0.4482	-0.844
Ngp	-1.17575	-1.22603	-1.2531	0.6838	0.34339	1.21877	1.06196	0.92987	1.05622	-0.96	-0.0132	-0.6657

Inpp4b	-1.08295	-1.31673	-1.0693	0.62855	0.82569	0.89053	1.04897	1.21308	0.92174	-0.859	-0.284	-0.9164
Ccr6	-1.11846	-1.09526	-1.1143	-0.1549	1.11125	0.78756	1.46047	1.11602	0.86864	-0.328	-0.6843	-0.8482
Il1r2	-1.62416	-1.24866	-1.3073	0.15313	0.81844	0.51242	1.34971	1.14407	0.90304	-0.163	-0.1664	-0.3708
Lag3	-1.56071	-1.33055	-1.2079	0.57414	0.72285	0.85788	1.07719	1.12093	0.90809	-0.254	-0.3338	-0.5738
Ramp3	-0.92711	-0.89398	-1.4547	0.40849	0.90313	0.77365	0.9506	0.94626	1.46743	-0.965	-0.726	-0.4825
Ikzf2	-1.17235	-1.32783	-1.2528	0.60494	0.8036	0.26987	1.22269	0.94474	1.42103	-0.616	-0.3514	-0.5464
Tox	-1.21898	-1.17724	-1.3072	0.75672	0.59713	0.72533	1.08735	1.19456	1.10781	-0.806	-0.3852	-0.5747
Napsa	-1.18527	-0.93334	-1.366	0.87322	0.69276	0.6271	1.15471	0.97555	1.2115	-0.626	-0.5339	-0.8902
Pacsin1	-1.23426	-1.32137	-0.7562	0.49221	0.73641	0.58837	1.33961	1.00704	1.15153	-0.088	-1.117	-0.7987
Ccrl2	-1.05982	-1.42802	-0.8589	0.48506	0.49641	0.7298	1.11156	1.25348	1.33181	-0.773	-0.8414	-0.4474
Penk	-1.13717	-1.11509	-1.1283	0.42844	0.58375	0.44112	1.45473	1.24568	1.20317	-0.559	-0.8482	-0.5689
Ociad2	-1.07172	-1.01223	-0.8481	0.42138	0.61615	0.51317	1.43783	1.02636	1.40823	-0.832	-1.0689	-0.5902
Rsad2	-1.59793	-1.2948	-0.4781	0.50911	0.87045	0.70398	1.75895	0.4828	0.66501	-1.047	-0.3018	-0.2712
Hba-a2	-2.20123	-0.80206	-0.0936	0.55973	0.53099	0.8197	1.4506	0.49471	0.45916	-1.203	-0.345	0.3302
Hbb-b1	-2.10343	-0.84966	-0.1304	0.62106	0.66185	0.8105	1.33782	0.64054	0.56	-1.222	-0.5475	0.2214
Hba-a1	-2.09961	-0.91112	-0.1506	0.59901	0.57948	0.85358	1.44884	0.55208	0.49499	-1.198	-0.414	0.2453
Hbb-bt	-2.15317	-0.77064	-0.1896	0.6498	0.60941	0.77234	1.43029	0.57042	0.51081	-1.202	-0.461	0.2333
Alas2	-1.71176	-0.88451	-0.336	0.79531	0.67534	0.99545	1.51014	0.6493	0.30889	-0.97	-1.1582	0.1257
Apol11b	-1.78153	-0.73728	-0.3002	0.82433	0.46237	1.36377	1.32895	0.67105	0.20558	-1.367	-0.545	-0.1253
Spp1	-0.98562	-0.9381	-0.9483	1.18639	-0.6053	0.63058	1.28991	1.08868	1.21691	-0.937	-0.9297	-0.068
Chil3	-1.13977	-1.02576	-0.8267	1.29392	0.46493	0.81368	1.26887	0.35354	1.17469	-1.011	-1.0971	-0.2695
Klra3	-1.00199	-0.50815	-0.8268	0.93047	0.3531	0.50072	1.39159	0.95903	1.29809	-1.251	-0.9691	-0.8755
Izumo1r	-0.98742	-1.08778	-0.7723	1.16001	0.60557	0.44519	1.19199	0.99632	1.18087	-0.766	-1.041	-0.9252
Chn2	-0.93231	-1.10508	-0.9531	0.9672	0.47989	0.78188	1.23386	1.01864	1.11533	-1.038	-0.5466	-1.022
Camp	-0.97164	-1.118	-1.158	1.2186	0.38299	0.81473	1.2443	0.6654	1.18521	-0.961	-0.6195	-0.6832
S100a8	-1.05386	-1.13828	-1.0218	1.14236	0.52668	0.66937	1.31047	0.72852	1.17652	-0.916	-0.8418	-0.5817
S100a9	-1.16952	-1.31918	-0.8569	1.07698	0.5432	0.62623	1.29028	0.78745	1.17919	-0.936	-0.6914	-0.5306
Il2ra	-0.15009	-0.06174	-0.0901	-1.0536	-1.093	-1.36286	-0.1269	-0.1058	-0.40502	1.4026	1.56651	1.4801
Il1rl1	-0.32107	0.18196	-0.0432	-1.5346	-1.1646	-1.1279	-0.1911	0.09806	-0.07408	1.3324	1.36249	1.4817
Prag1	-0.61348	-0.37344	-0.3869	-0.7139	-1.0275	-1.54909	0.34318	0.09894	-0.04588	1.4369	1.70472	1.1264
Pus7	-0.49828	-0.51313	-0.7158	-0.3459	-0.6709	-1.83197	-0.1469	0.51388	0.11381	1.2262	1.69163	1.1775
Cd40lg	-0.73418	-0.51408	-0.8025	-1.1298	-0.8345	-0.45724	-0.2053	0.19698	-0.16956	1.5205	1.72553	1.4042
Sphk1	-0.767	-0.77324	-0.7628	-0.7706	-0.7318	-0.77245	-0.2866	0.22271	-0.0507	1.7046	1.46292	1.525
Furin	-0.83923	-0.73836	-0.7974	-0.8742	-0.7083	-0.79357	0.0256	0.08053	0.00286	1.413	1.64251	1.5866
Zbtb32	-0.81461	-0.91022	-1.0033	-0.6857	-0.6415	-0.70912	-0.0736	0.1187	0.11435	1.4335	1.59233	1.5792
Lta	-0.35975	-0.8953	-0.4954	-1.2616	-0.4986	-0.66394	-0.3596	0.01351	-0.17455	1.4153	1.62592	1.6541
Mpzl2	-0.64744	-0.66223	-0.6568	-0.6609	-0.6398	-0.66084	-0.1243	-0.1705	-0.65371	1.513	1.7546	1.6089
Ccl9	-0.47675	-0.56983	-0.564	-0.7209	-1.1612	-0.81966	0.01567	-0.3812	0.0127	1.2148	1.8157	1.6346
Jaml	-0.21853	-0.8232	-0.5529	-1.1711	-0.7052	-1.16861	0.02774	0.03858	0.08736	1.6007	1.45526	1.4299
Rhbdf2	-0.32521	-0.46756	-0.8863	-0.9711	-0.7087	-1.28029	0.20445	0.11301	-0.16256	1.5744	1.5345	1.3753
Ctps	-0.39394	-0.77638	-1.6043	-0.4806	-0.9329	-0.90012	0.2451	0.55998	0.36787	1.0119	1.51848	1.3849
Polr1b	-0.51416	-0.4196	-1.4268	-0.6692	-0.7046	-1.14221	0.34183	0.36775	-0.0677	1.3383	1.57589	1.3205
Slc41a1	-0.96495	-1.20267	-1.2485	-0.4946	-0.782	-0.57312	0.74157	0.59039	0.07389	1.3629	1.25633	1.2408
Sema7a	-1.22017	-1.09913	-1.2361	-0.3592	-0.8838	-0.3043	0.33972	0.27535	0.55126	1.3664	1.51751	1.0524
Zswim4	-0.84001	-1.27207	-0.7355	-0.7714	-1.0026	-0.74627	0.31213	0.68995	0.40576	1.4108	1.28247	1.2668
Mfsd2a	-0.9852	-0.90396	-1.0664	-1.0676	-1.0321	-0.4341	0.48233	0.78515	0.53968	1.1664	1.34713	1.1687
Srm	-1.06813	-1.10923	-0.9836	-1.0468	-0.7713	-0.48738	0.64247	0.47912	0.58132	1.1351	1.38438	1.2441
Pim3	-1.26035	-0.97358	-0.8349	-0.7403	-1.0481	-0.75875	0.86731	0.66432	0.68461	1.0442	1.08487	1.2707
Marcksl1	-1.24565	-0.86248	-0.8419	-0.7474	-1.0831	-0.75016	0.63999	0.66035	0.58307	1.0616	1.4438	1.1419
Ccdc86	-0.84796	-0.8268	-0.7545	-0.8318	-1.2341	-0.92487	0.47775	0.5225	0.52214	1.0205	1.48256	1.3945

Spry1	-0.99688	-0.70924	-0.7921	-0.9768	-0.958	-1.03129	0.69071	0.5034	0.39967	1.2451	1.4754	1.1501
Trib1	-1.0882	-0.79309	-0.8302	-0.9797	-0.7816	-0.92755	0.61095	0.33712	0.44392	1.2424	1.46659	1.2993
Rgcc	-1.07129	-1.04827	-0.8723	-0.7571	-0.8378	-0.91574	0.72037	0.43087	0.51985	1.2466	1.36044	1.2244
Ifng	-0.94018	-0.94266	-0.9039	-0.9057	-0.8634	-0.95804	0.60907	0.49848	0.52291	1.2294	1.34962	1.3043
Cish	-0.94868	-1.10863	-0.8203	-0.8113	-0.8811	-0.98542	0.6275	0.62553	0.54224	1.2681	1.26187	1.2301
Ccl1	-0.87473	-0.96997	-0.9565	-0.9834	-0.9281	-0.92004	0.78317	0.5616	0.71608	1.135	1.26895	1.1679
Ppan	-0.93559	-0.91685	-0.7654	-1.0202	-1.0055	-0.90595	0.71489	0.58724	0.49841	1.1026	1.36789	1.2785
Slc7a1	-0.86266	-0.92148	-0.8688	-0.8819	-1.0854	-0.92883	0.8422	0.5352	0.46261	1.2473	1.36901	1.0926
Sh2b3	-0.27703	-0.40491	-0.7015	-1.9256	-0.6721	-0.44647	-0.0559	0.59828	-0.17498	1.4256	1.23656	1.3982
Atad3a	-0.84254	-0.69122	-1.0235	-1.6282	-0.2653	-0.52296	0.31638	0.2686	0.37535	1.1012	1.49803	1.4142
Pprc1	-0.52693	-0.91919	-0.6164	-1.8914	-0.6875	-0.29288	0.25246	0.71298	0.26767	1.0507	1.55027	1.1002
Fhl2	-0.42491	-0.51951	-0.1799	-1.3404	-1.1341	-1.59659	0.86396	0.45115	0.67364	0.9189	1.04412	1.2437
Map3k8	-0.58737	-0.31815	-0.5198	-1.3816	-1.0991	-1.40785	0.6466	0.97165	0.37784	1.079	1.05087	1.1879
Il2	-0.86315	-0.81429	-0.899	-0.8749	-0.8347	-0.90707	0.42551	0.28689	0.16888	1.3572	1.48243	1.4721
Phlda1	-0.97544	-0.89435	-0.7821	-1.1608	-0.7029	-0.77463	0.39718	0.26652	0.48807	1.3356	1.49287	1.3099
Utf1	-1.04677	-0.97934	-0.8609	-1.0535	-0.6815	-0.779	0.44904	0.36296	0.61303	1.1963	1.48803	1.2916
Tnf	-0.85588	-0.99405	-0.9058	-0.9691	-0.8117	-0.83126	0.4266	0.47968	0.35094	1.4009	1.38068	1.3289
Tnfsf9	-1.00983	-0.99399	-0.8228	-1.0394	-0.7742	-0.72908	0.33391	0.58049	0.40836	1.3302	1.48073	1.2356
Fosl1	-0.76017	-0.73328	-0.8705	-0.9326	-0.8878	-0.91464	-0.1252	0.52663	0.41451	1.2406	1.52225	1.5202
Slco4a1	-0.71026	-0.95293	-0.8533	-0.7798	-0.925	-1.11926	0.39468	0.58441	0.27643	1.3752	1.41026	1.2997
Slamf1	-0.71535	-0.75896	-0.9313	-0.6742	-0.8438	-1.26088	0.08623	0.57673	0.34061	1.333	1.42928	1.4187
Shmt1	-1.01397	-0.6575	-0.5829	-1.4592	-0.9233	-0.72626	0.67411	0.48094	0.40495	1.1557	1.3417	1.3057
Bcat1	-0.91003	-0.6821	-0.6619	-1.4493	-0.6884	-1.00293	0.88527	0.45618	0.3512	1.1794	1.25527	1.2675
Grwd1	-1.09871	-0.61401	-0.6143	-1.3817	-0.7501	-0.97644	0.83174	0.49533	0.47919	1.0317	1.36598	1.2313
Atf3	-0.97781	-0.81128	-0.3619	-1.0163	-1.0518	-1.10832	0.56354	0.20004	0.69903	1.1198	1.47122	1.2738
Hk2	-0.63359	-0.70628	-0.4159	-1.3269	-1.113	-0.8689	0.30592	0.26355	0.31384	1.169	1.56631	1.446
Grasp	-0.78548	-0.6524	-0.5596	-1.2111	-1.0745	-0.99595	0.59583	0.42427	0.22914	1.233	1.32169	1.4752
Cdc14a	-0.5068	-1.0703	-0.8406	-0.98	-1.3684	-0.80918	0.99179	1.25711	1.01314	0.8968	0.65694	0.7596
Sult2b1	-0.95905	-0.89414	-1.015	-1.1899	-0.8189	-0.69776	1.31344	1.11989	1.18319	0.5465	0.66977	0.742
Btg3	-0.87244	-1.08721	-0.9187	-0.7327	-1.0546	-0.90547	1.46646	1.17112	0.89428	0.5542	0.66381	0.8212
Gfod1	-0.94056	-1.16676	-0.3272	-1.2451	-0.9216	-0.83557	1.13364	0.32629	1.01523	1.1833	1.15891	0.6194
Trmt61a	-0.78749	-0.89731	-0.7268	-0.963	-0.4341	-1.55416	0.75371	0.33063	0.76724	0.9243	1.13757	1.4494
Spred1	-0.86759	-0.89713	-0.4041	-1.3437	-0.6299	-1.33689	1.19814	1.05897	1.00208	0.5643	0.68861	0.9673
Hilpda	-1.26031	-0.85804	-0.5471	-0.8567	-0.6608	-1.38459	1.0624	0.92785	1.07286	0.7744	0.70484	1.0253
Btla	-0.9252	-0.8862	-0.9385	-1.6073	-0.429	-0.55421	1.15885	0.62873	0.39542	0.708	1.16182	1.2875
Fos	-1.01938	-1.18105	-0.7074	-1.0449	-0.761	-0.91752	0.64739	1.25249	0.74144	1.1219	1.00984	0.8582
Nab2	-1.05806	-1.05236	-0.9151	-1.4049	-0.5233	-0.59156	0.68041	1.02586	0.65528	1.0592	0.97766	1.1469
Tnfsf14	-0.98695	-1.16402	-1.0614	-0.8105	-0.7685	-0.84631	0.67328	0.84959	0.68075	1.1618	1.20889	1.0634
Nfkbid	-0.98861	-0.99872	-1.0091	-0.8646	-0.7907	-0.97197	0.64581	0.74759	0.63436	1.2214	1.18094	1.1936
Csf2	-0.89729	-0.99232	-1.0079	-0.9565	-0.6983	-1.00439	0.62687	0.72533	0.52064	1.0287	1.39739	1.2578
Egr3	-1.03883	-0.93606	-0.9136	-1.0451	-0.7881	-0.94176	0.65098	0.89554	0.74581	1.0061	1.27962	1.0853
Crtam	-1.04928	-0.8794	-0.9814	-0.9036	-0.8553	-0.99545	0.81847	0.75928	0.65501	1.0223	1.22727	1.1819
Tagap	-0.90887	-1.00478	-1.2109	-0.8904	-0.92	-0.74762	0.83414	0.97272	0.71229	0.9642	1.0643	1.1349
Fosl2	-0.88083	-1.10089	-1.1191	-0.9747	-0.843	-0.73381	1.06907	0.83309	0.55084	1.1288	1.07087	0.9997
Plk3	-0.88616	-1.00757	-0.9808	-0.8048	-1.2114	-0.76665	0.70002	0.88285	0.72563	1.1143	1.11586	1.1188
Nr4a1	-1.02183	-1.02008	-0.8832	-1.033	-0.881	-0.85516	0.75814	0.87507	0.74852	1.1413	1.10359	1.0675
Irf4	-0.934	-0.98675	-0.8969	-1.1302	-0.914	-0.78372	0.70844	0.76238	0.71484	1.3088	1.04408	1.107
Myc	-1.22567	-1.14174	-1.1915	-0.7337	-0.7211	-0.57751	0.75523	0.79356	0.75013	1.0298	1.13261	1.1299
Gnl3	-1.25842	-1.14424	-1.0188	-0.6052	-0.6678	-0.87231	0.79412	0.66941	0.65692	1.0158	1.21075	1.2198
Nr4a3	-1.18054	-1.19083	-0.9474	-0.7162	-0.83	-0.77542	0.83727	0.77807	0.78002	1.1151	1.23983	0.8901

Kdm6b	-1.10232	-1.44895	-0.9194	-0.7093	-0.5916	-0.78299	0.93635	0.56517	0.85778	1.1057	1.17964	0.9099
Tnfsf11	-1.09118	-1.15503	-1.1876	-0.8893	-0.3966	-0.87574	0.85126	0.83608	0.81894	0.8297	1.21352	1.046
Rel	-1.10155	-1.17119	-1.2332	-0.7149	-0.6067	-0.80468	0.84833	1.09713	0.91618	0.7946	1.09125	0.8847
Cd160	-1.52912	-1.01865	-1.1061	-0.541	-0.5761	-0.76819	1.09421	0.72793	0.88907	0.9211	0.9842	0.9227
Gch1	-1.27568	-1.0473	-1.1354	-0.7083	-0.7994	-0.68393	1.01717	0.90594	1.11301	0.8146	0.92873	0.8706
Tfrc	-1.17969	-1.05595	-1.2304	-0.6069	-0.7891	-0.78051	1.03358	0.86411	0.79049	0.846	1.03288	1.0755
Eef1e1	-1.28167	-0.65678	-0.652	-0.7479	-1.0403	-1.2237	1.16512	0.82469	0.80721	0.8886	1.10988	0.8069
Xcl1	-1.07249	-1.06903	-1.0713	-0.7815	-0.7819	-0.92175	0.93363	0.82838	0.78297	1.0716	1.07494	1.0064
Orai1	-0.94897	-1.03502	-0.9554	-0.7619	-0.9113	-1.09613	0.87275	0.80907	0.8596	1.0056	1.07173	1.09
Irf8	-1.10653	-0.96049	-1.0401	-0.8897	-0.8521	-0.86377	1.08447	0.99706	1.05479	0.7757	0.86943	0.9312
Egr2	-1.06416	-1.02219	-0.942	-0.7699	-0.8752	-1.04626	0.99762	1.08805	0.89026	0.9234	0.93174	0.8886
Ccl3	-0.99917	-0.97286	-0.9435	-0.9956	-0.8744	-0.95438	1.00154	0.92443	0.9749	0.9476	0.98747	0.904
Ccl4	-0.9839	-0.96478	-0.9169	-0.9873	-0.8847	-1.00141	1.01804	0.89784	0.98929	0.9295	0.98123	0.9231
Socs1	-0.99774	-0.92573	-0.7916	-0.7855	-1.0159	-1.13417	0.68653	0.85833	0.8959	0.7695	1.16991	1.2704
Pdcd1lg2	-0.94586	-1.09963	-0.8195	-0.9568	-0.8047	-1.04816	0.88864	0.9275	1.15904	0.6117	1.13386	0.9539
Arl5b	-0.85061	-1.16918	-0.6442	-0.8631	-1.0938	-1.05597	0.94637	0.978	1.08418	0.8454	1.04222	0.7807
Il21	-1.27982	-1.15439	-1.2173	-0.3469	-0.3529	-0.53827	1.30244	1.52805	1.33498	0.2651	0.33933	0.1196
Gpd2	-1.08823	-0.96746	-1.3684	-0.5423	-0.7984	-0.29124	1.45005	1.41161	1.22989	0.1763	0.5376	0.2506
Spin4	-0.9668	-1.08593	-1.0545	-0.3723	-0.4751	-1.11446	1.2035	1.57118	1.292	-0.069	0.49773	0.5736
Gpr65	-1.2422	-0.85103	-0.7137	-0.7688	-0.6007	-1.03691	1.60263	1.24706	1.30153	0.3126	0.37946	0.3701
Rgs1	-1.51094	-1.04218	-0.8968	-0.516	-0.5045	-0.86689	1.28645	1.24106	1.23762	0.4767	0.55653	0.539
Srxn1	-1.68003	-0.9054	-1.1744	-0.6714	-0.4976	-0.45661	1.08509	1.19279	0.95295	0.626	0.80502	0.7235
Dusp6	-1.26505	-1.09166	-1.2588	-0.6846	-0.9489	-0.22948	1.06135	1.12231	1.04359	0.9078	0.6682	0.6752
Nr4a2	-1.29953	-1.33895	-1.1691	-0.5717	-0.5859	-0.51447	1.07711	1.15646	1.06119	0.7551	0.73924	0.6904
Rgs16	-1.31213	-1.29113	-1.2387	-0.6284	-0.5255	-0.4379	1.09905	1.19421	1.07553	0.6652	0.72229	0.6775
Il10	-1.16857	-1.10108	-1.137	-0.6624	-0.7736	-0.73507	1.05446	1.25298	1.1801	0.7924	0.58835	0.7095
Gem	-1.28159	-1.0053	-1.2758	-0.5127	-0.7183	-0.72723	1.18529	1.20151	1.02534	0.5653	0.74928	0.7942
Dusp4	-1.19792	-0.95576	-1.2157	-0.8838	-0.8486	-0.52195	0.97831	1.21692	0.94682	0.778	0.78456	0.9191
Egr1	-1.14835	-1.02634	-0.8885	-1.0219	-0.794	-0.79694	1.06947	1.21144	0.98792	0.756	0.8839	0.7672
Ier2	-1.2837	-1.05158	-0.9477	-0.8942	-0.7836	-0.6495	1.10229	1.2535	0.98756	0.6069	0.88018	0.7798
Hivep3	-1.06389	-1.13362	-1.072	-0.8149	-0.9682	-0.60403	0.98047	0.7281	0.98374	0.8929	1.21682	0.8547
Zfp36l1	-1.19277	-1.18077	-0.9389	-0.8084	-0.8142	-0.65363	1.21721	0.90659	1.21047	0.7299	0.9793	0.5452
Chst2	-1.21223	-1.20155	-1.2041	-1.16	-0.139	-0.2444	1.05183	1.20099	1.22913	0.191	0.67934	0.8091
Eea1	-1.39279	-1.18491	-1.3787	-0.6206	-0.1586	-0.37025	1.22882	1.12288	1.28382	0.5057	0.66652	0.2981
Tnfrsf9	-1.5397	-1.40364	-1.2138	-0.2512	-0.2349	-0.5352	1.15503	0.94194	1.04692	0.4437	0.76847	0.8224
Mdfic	-1.33366	-1.40359	-1.6163	-0.2096	-0.2787	-0.24156	0.93665	0.98338	1.03225	0.6423	0.86304	0.6258
Tnfsf8	-1.57395	-1.6377	-1.5802	0.15311	0.36503	0.09181	0.96788	0.83076	0.63132	0.3309	0.84048	0.5806
Trps1	-1.21936	-1.304	-1.3559	0.28717	0.05447	0.11684	1.45841	1.11503	1.48536	-0.386	0.11674	-0.3687
Tnfrsf4	-1.42009	-1.45284	-1.3621	0.29363	0.46433	0.27582	1.30196	1.12494	1.16677	-0.551	0.05603	0.1026
Ptger2	-1.2337	-1.50767	-1.1747	0.04477	0.19879	-0.22116	1.45397	1.331	1.23997	-0.475	0.28657	0.0576
Plscr1	-1.69211	-1.20164	-1.3768	-0.0499	0.00682	0.00672	1.23956	1.24458	1.17841	-0.111	0.48532	0.2696
Hic1	-1.17647	-1.3787	-1.6121	0.15399	-0.0374	-0.07793	1.43283	1.22378	1.21813	0.1609	-0.0543	0.1473
Slc37a2	-1.49641	-1.37257	-1.5594	0.31463	0.46815	0.22854	1.24119	1.2462	0.95113	0.1754	0.01633	-0.2133
Gcnt1	-0.6889	-1.05063	-1.0027	-1.1258	0.16677	0.71109	1.41851	0.89466	1.37792	-1.292	0.12032	0.4704
Calcb	-0.81207	-0.82111	-0.7	-0.016	-0.0234	-0.05945	1.70805	1.41121	1.59104	-0.75	-0.7894	-0.7389
Adrb1	-0.58998	-1.32097	-1.0717	0.00148	-0.2285	0.55932	1.34062	1.4401	1.54724	-0.752	-0.722	-0.2036
Spry2	-1.02764	-1.06331	-0.9299	-0.4023	0.1233	-0.09702	1.65732	1.49011	1.44099	-0.739	-0.4125	-0.0395
Srgap3	-1.07801	-1.08502	-1.0733	-0.2145	0.4909	0.55383	1.50034	1.07601	1.53297	-0.923	-0.3941	-0.3863
Flt1	-1.09135	-1.03862	-1.0685	0.3029	-0.044	-0.482	1.65808	1.64897	0.80255	-1.041	0.22708	0.1255
P3h2	-0.67941	-0.79958	-0.7271	-0.3766	-0.73	-0.74283	1.98686	1.28184	1.3513	-0.771	0.33855	-0.1315

Figure 2b

Frequency of Nr4a1-GFP+ cells (out of P14 cells)

dpi	1	2	3	7	14	21	28
mouse_1	83.94	68.6	49.2	47	24.5	23.3	4.25
mouse_2	90.32	75	47.1	55.2	27.5	25.7	3.32
mouse_3				36.2	28.8	13.5	1.99

Figure 2c

Expression of Nr4a1-GFP (normalized medians) in Nr4a1-GFP+ cells

dpi	1	2	3	7	14	21	28
mouse_1	86.11	16.53	15.522	10.953	8.14	2.41	1.847
mouse_2	83.8	17.67	15.504	12.512	8.3	2.3	1.989
mouse_3				10.113	8.57	1.79	1.96
mouse_4						2.7	1.605

Figure 2d

Expression of Nr4a1-GFP (normalized medians) in Nr4a1-GFP+ cells

	blood	spleen	BM	LN	lung	liver	kidney	d1 (spleen)
mouse_1	9.1	13.533	11.033	10.567	10.7	9.533	9.9333	85.58207
mouse_2	10.667	10.4	9.3	10.4	11.033	9.667	10.4	83.80058
mouse_3	11.2	12.6	9.2667	11.067	11.333	9.567	10.567	
mouse_4	12.2	13.067	8.9667	11.567	11.667	9.267	10.267	

Figure 3

PD-1 medians

	acute degr+	chronic degr+	acute degr-	chronic degr-
mouse_1	679	7269	565	3761
mouse_2	538	8171	477	5824
mouse_3	568	8467	506	2911

Nr4a1-GFP medians

	acute degr+	chronic degr+	acute degr-	chronic degr-
mouse_1	5103	3151	750	444
mouse_2	4841	3539	1040	534
mouse_3	4623	2040	980	624

CD107a medians

	acute degr+	chronic degr+	acute degr-	chronic degr-
mouse_1	12698	8208	28.2	62.9
mouse_2	12101	6226	57.8	108
mouse_3	11854	6363	88.7	77.1

IFNg medians

	acute degr+	chronic degr+	acute degr-	chronic degr-
mouse_1	3539	1080	135	266
mouse_2	3476	556	171	263
mouse_3	2712	480	202	217

Figure 4c

Nr4a1-GFP medians of primed cells

	BM	kidney	LN	liver	lung	blood	spleen
mouse_1	43.45699	66.71423	66.42886	51.54912	62.96372	31.00285	65.91928
mouse_2	50.75418	65.12434	66.97921	54.54545	64.34978	38.34081	69.56788
mouse_3	49.40889	65.6543	65.51162	56.93029	58.86669	35.69099	71.28007

Figure 5c

	BM	blood	kidney	LN	liver	lung	spleen
mouse_1	0.52439	0.75	0.577231	0.462842	0.583009	0.521053	0.447356
mouse_2	0.455598	0.642202	0.55334	0.462264	0.573858	0.502577	0.390244
mouse_3	0.429268	0.82904	0.613648	0.526171	0.62273	0.578358	0.425926

Figure 5d

	BM	blood	kidney	LN	liver	lung	spleen
mouse_1	0.252101	0.2	0.914236	0.272629	0.918818	0.786601	0.418118
mouse_2	0.086957	0.230263	0.890716	0.304124	0.910936	0.796117	0.384314
mouse_3	0.093851	0.5	0.899001	0.373786	0.931784	0.707418	0.497253

Figure 6c

	αPD-L1	ctr
mouse_1	26.4	5.45
mouse_2	28	22.5
mouse_3	34.2	17.8

Figure 6d

	αPD-L1	ctr
mouse_1	9.664727	7.780549
mouse_2	8.888889	7.337213
mouse_3	9.598227	8.379052

Figure 6e

	αPD-L1	ctr
mouse_1	4913	2319
mouse_2	3959	3214
mouse_3	4057	2843

Figure 7c

	pulsed	unpulsed
mouse_1	0.039027	1.04831
mouse_2	0.002074	0.958852
mouse_3	0.006305	1.038819
mouse_4	0.061505	0.954018

Figure 7e

	unpulsed	pulsed
mouse_1	290	406
mouse_2	306	312
mouse_3	278	378
mouse_4	289	392

Figure 7f

	unpulsed	pulsed
mouse_1	12.2	16.5
mouse_2	10.8	19.1
mouse_3	6.45	18.7
mouse_4	10.2	22.5

Figure 7g

	blood	spleen	BM	LN	lung	liver	kidney
mouse_1	7.95	3.5101	4.0988	3.12	4.19	4.27	7.635
mouse_2	7.357	3.1206	3.5598	3.04	4.29	4.48	6.944
mouse_3	9.465	3.4712	4.5276	3.44	4.19	4.88	7.022

Supplementray Figure 1a
GFP median after in vitro stimulation

hours	mouse 1	mouse 2
0	112	95.1
2.2	1842	1509
4.516667	2136	1828
5.766667	1906	2006
20.76667	2193	2219
24.26667	2136	2107
29.26667	762	848
39.6	685	703
53.76667	463	455
65.26667	233	236
71.85	204	207
96.65	156	148

Supplementray Figure 1d

Nr4a1-GFP frequency

2w

	blood	spleen	BM	LN	lung	liver	kidney
mouse_1	6.04	32.6	53.1	66.5	41.6	20.1	30.1
mouse_2	4.17	48.3	56.9	58.2	52	29.8	26.7
mouse_3	11.1	50.7	51	31.4	44	30.2	29.3

3w

	blood	spleen	BM	LN	lung	liver	kidney
mouse_1		23.3	13.6	32.9	34.3	3.33	6.54
mouse_2	0	25.7	14.6	33.8	40.7	2.54	12.4
mouse_3	2.44	13.5	8.16	24.3	41.2	1.32	11.7
mouse_4	3.6	26.8	16	44.1	44.7	10.3	14.8

4w

	blood	spleen	BM	LN	lung	liver	kidney
mouse_1	1.1	15	4.29	28.9	26.3	2.39	16
mouse_2	1.44	18.7	9.12	5.13	22.9	2.93	5.68
mouse_3	4.11	17	6.88	15.2	33.5		9.85
mouse_4	4.84	7.66	4.27	11.2	37.1		6.03

Nr4a1-GFP medians

2w

	blood	spleen	BM	LN	lung	liver	kidney
mouse_1	5.340909	5.909091	6.548295	8.75	6.676136	5.340909	6.25
mouse_2	6.09375	6.775568	6.448864	8.636364	6.931818	5.653409	6.71875
mouse_3	5.099432	6.71875	6.775568	7.855114	6.960227	5.752841	6.221591

3w

	blood	spleen	BM	LN	lung	liver	kidney
mouse_1	4.900568	6.363636	5.113636	7.244318	6.903409	6.903409	6.448864
mouse_2		7.159091	5.568182	8.565341	6.676136	7.272727	6.193182
mouse_3	6.065341	6.065341	4.943182	6.832386	7.201705	5.454545	5.142045
mouse_4	5.667614	5.696023	5.525568	7.642045	6.960227	5.355114	5.653409

4w

mouse_1	blood	spleen	BM	LN	lung	liver	kidney
mouse_2	4.346591	6.448864	6.392045	6.875	6.676136	5.397727	5.909091
mouse_3	5.042614	5.965909	5.525568	7.017045	6.40625	4.673295	5.042614
mouse_4	6.875	7.670455	5.78125	6.207386	7.059659		5.113636
	5.298295	5.809659	5.411932	6.576705	6.633523		5.696023

Supplementray Figure 2

Nr4a1-GFP medians

tissue	blood			
	acute degr+	chronic degr+	acute degr-	chronic degr-
mouse_1	5640	2168	255	134
mouse_2	4872	2539	301	249
mouse_3	5202	2231	244	211
	BM			
	acute degr+	chronic degr+	acute degr-	chronic degr-
mouse_1	6777	2266	360	244
mouse_2	6703	1860	204	355
mouse_3	6133	3157	265	153
	LN			
	acute degr+	chronic degr+	acute degr-	chronic degr-
mouse_1	7562	2391	387	356
mouse_2	7414	2227	454	427
mouse_3	6733	1920	490	468
	lung			
	acute degr+	chronic degr+	acute degr-	chronic degr-
mouse_1	7317	1591	399	383
mouse_2	6200	3476	384	320
mouse_3	6733	1758	358	377
	liver			
	acute degr+	chronic degr+	acute degr-	chronic degr-
mouse_1	6777	1784	506	202
mouse_2	7284	2160	464	355
mouse_3	5849	1976	421	199
	kidney			
	acute degr+	chronic degr+	acute degr-	chronic degr-
mouse_1	7704.488	1156.33	453.334	438.0038
mouse_2		2809.795		429.2438
mouse_3	8705.326	1984.157	435.8138	310.9827

CD107a medians

tissue	blood			
	acute degr+	chronic degr+	acute degr-	chronic degr-
mouse_1	10895	10870	48.8	50.1
mouse_2	10772	9280	68.1	55.2
mouse_3	10846	10483	62.9	51.4
	BM			
	acute degr+	chronic degr+	acute degr-	chronic degr-
mouse_1	11746	9642	39.8	113
mouse_2	12582	7765	62.9	103

mouse_3	13388	15241	73.2	126

LN

	acute degr+	chronic degr+	acute degr-	chronic degr-
mouse_1	12934	7381	66.8	80.9
mouse_2	8244	4841	123	53.9
mouse_3	13419	4751	121	57.8

lung

	acute degr+	chronic degr+	acute degr-	chronic degr-
mouse_1	11171	5616	55.2	38.5
mouse_2	11908	4721	59.1	23.1
mouse_3	11827	3259	55.2	42.4

liver

	acute degr+	chronic degr+	acute degr-	chronic degr-
mouse_1	11800	9664	88.7	162
mouse_2	12073	8913	113	38.5
mouse_3	10945	7852	92.5	39.8

kidney

	acute degr+	chronic degr+	acute degr-	chronic degr-
mouse_1	6097.208	6714.117	84.45053	161.7138
mouse_2		9331.484		107.8092
mouse_3	9331.484	16339.08	92.23675	76.66431

IFNg medians

tissue blood

	acute degr+	chronic degr+	acute degr-	chronic degr-
mouse_1	1312	376	187	245
mouse_2	1057	617	156	200
mouse_3	1285	347	181	216

BM

	acute degr+	chronic degr+	acute degr-	chronic degr-
mouse_1	2447	383	171	229
mouse_2	2509	434	181	285
mouse_3	2894	343	230	170

LN

	acute degr+	chronic degr+	acute degr-	chronic degr-
mouse_1	3004	1086	224	278
mouse_2	3547	1114	229	261
mouse_3	3345	962	254	229

lung

	acute degr+	chronic degr+	acute degr-	chronic degr-
mouse_1	2359	1006	178	213
mouse_2	1784	1453	190	211

mouse_3	1609	556	170	195

liver

	acute degr+	chronic degr+	acute degr-	chronic degr-
mouse_1	3634	458	212	258
mouse_2	3208	496	185	275
mouse_3	2804	510	258	221

kidney

	acute degr+	chronic degr+	acute degr-	chronic degr-
mouse_1	1086.386	246.1506	217.0695	267.9614
mouse_2		475.6834		273.1544
mouse_3	1561.031	580.583	184.8726	201.4903

PD1 medians

tissue blood

	acute degr+	chronic degr+	acute degr-	chronic degr-
mouse_1	817	8716	692	9449
mouse_2	671	10435	610	7269
mouse_3	652	10364	594	5713

BM

	acute degr+	chronic degr+	acute degr-	chronic degr-
mouse_1	868	10087	597	7817
mouse_2	851	11693	625	10225
mouse_3	755	13544	775	10506

LN

	acute degr+	chronic degr+	acute degr-	chronic degr-
mouse_1	869	5925	750	4955
mouse_2	912	6573	952	5427
mouse_3	722	6674	817	5257

lung

	acute degr+	chronic degr+	acute degr-	chronic degr-
mouse_1	634	5862	717	4604
mouse_2	685	5257	690	2624
mouse_3	544	4903	646	3671

liver

	acute degr+	chronic degr+	acute degr-	chronic degr-
mouse_1	633	8208	391	6390
mouse_2	625	8355	460	5370
mouse_3	516	4903	493	3671

kidney

	acute degr+	chronic degr+	acute degr-	chronic degr-
mouse_1	1071.574	7250.985	642.9445	4912.232
mouse_2		6636.956		3951.217

mouse_3	920.193	7371.75	534.0862	2639.814

Supplementray Figure 3a

	blood	spleen	BM	LN	lung	liver	kidney
mouse_1	1.13	15.8	1.79	6.8	33.2	11.9	13.8
mouse_2	3.07	15.9	2.1	9.48	30.6	12.7	14.5
mouse_3	4.11	22	1.81	9.56	18.2	13.3	15.1

Supplementray Figure 3b

	blood	spleen	BM	LN	lung	liver	kidney
mouse_1	75.7	44.9	50.3	20.2	55.2	73.9	73.2
mouse_2	66.1	38.4	42.9	19.6	53	74.8	69.6
mouse_3	83.6	41.9	41	26.1	59.6	78.5	71

Supplementray Figure 3c

PD-1 frequency

2w

	blood	spleen	BM	LN	lung	liver	kidney
mouse_1	99.6	99.6	99.6	97.3	99.1	99.9	99.9
mouse_2	98.6	99.6	100	95.6	98	99.8	99.3
mouse_3	100		93.3	58	73	86.7	97.1
mouse_4	99.7	99.5	99.6	88.6	98	99.9	99.9

3w

	blood	spleen	BM	LN	lung	liver	kidney
mouse_1		97.8	99	90.2	95.8	95	98
mouse_2	100	97	98.1	84	94.4	100	99.3
mouse_3	92.7	97.3	100	80.3	96.8	99.6	99.7
mouse_4	99.1	97.6	99.6	93.9	95.6	99.6	99.5

4w

	blood	spleen	BM	LN	lung	liver	kidney
mouse_1	98.1	94.9	100	95.6	92.7	99.4	98.4
mouse_2	99.5	99.5	100	71.8	95.5	99.8	94.8
mouse_3	98.6	98.5	99.4	79.7	94		97.6
mouse_4	96.8	98.9	100	78.9	92.7		95.8

PD-1 CD39 TIM-3 frequency

2w

	blood	spleen	BM	LN	lung	liver	kidney
mouse_1	90.636	71.9112	64.0428	59.2557	76.4061	79.6203	72.1278
mouse_2	88.1484	69.1224	69.5	63.3828	78.204	82.2352	74.5743
mouse_3	91.8		46.65	16.008	55.553	53.3205	64.7657
mouse_4	93.2195	72.436	69.5208	66.45	73.402	76.8231	66.6333

3w

	blood	spleen	BM	LN	lung	liver	kidney
mouse_1		62.2008	61.281	73.7836	80.7594	63.365	63.994
mouse_2	54.6	65.281	67.9833	62.328	83.4496	45.8	71.6946
mouse_3	43.9398	64.8018	60.4	50.2678	75.6976	55.1784	72.8807
mouse_4	68.7754	56.5104	67.5288	52.0206	74.8548	50.6964	63.3815

4w

	blood	spleen	BM	LN	lung	liver	kidney
mouse_1	43.0659	51.4358	36.4	62.8092	63.1287	29.5218	55.596
mouse_2	46.168	68.058	42.8	33.3152	50.5195	35.6286	60.198
mouse_3	45.1588	55.948	39.0642	33.3943	59.972		53.9728
mouse_4	54.8856	64.8784	51	43.9473	71.379		62.3658

Supplementray Figure 4a

	no blockade	αPD-L1
mouse_1	0.8	33.3
mouse_2	0.78	23.6
mouse_3	0.54	27.7
mouse_4	1.24	42.8

Supplementray Figure 4b

	αPDL1				ctr		
mouse	1	2	3	4	1	2	3
BM	6.01	6.49	15.5	11.2	4.89	6.83	6.3
blood	10.5	4.94	4.14	5.79	0.28	2.18	2.67
kidney	26.1	29.6	31.7	35.5	11.6	22.7	23
LN	54.1	41.2	39	54.2	24.9	31.5	23.4
liver	5.9	4.62	8.43	4.01	0.68	1.79	0.77
lung	44.8	33.9	41.4	51.4	33.6	37.5	32.7
spleen	24	16.3	29.4	21.6	7.19	16.4	17.2

Supplementray Figure 4c

	αPDL1				ctr		
mouse	1	2	3	4	1	2	3
BM	394	384	427	413	405	413	398
blood	460	366	424	538	535	546	465
kidney	502	483	481	458	370	402	431
LN	630	487	500	565	486	474	434
liver	440	474	409	387	389	374	434
lung	567	528	518	543	507	507	543
spleen	532	507	518	426	448	491	537

Supplementray Figure 4d

	αPDL1				ctr		
mouse	1	2	3	4	1	2	3
BM	30743	38910	34497	30160	25641	24683	32099
blood	7479	11429	17449	10945	21260	13327	22932
kidney	36110	35421	37892	36812	27280	29236	30597
LN	32099	21010	25824	20862	17946	16382	15100
liver	26321	26573	26196	26010	15348	21411	19165
lung	15312	10970	16004	19761	19948	14054	14283
spleen	19347	19854	24801	19995	13235	12073	10821

Supplementray Figure 5c

	αPDL1			co-blockade			ctr		
mouse	1	2	3	1	2	3	1	2	3
BM	6.44	6.31	5.18	5.28	8.63	3.92	2.64	4.41	2.91
blood	7.08	18.5	24.6	29.5	27	18.4	3	2.33	6.9
Kidney	25.5	30.6	40	42.2	34.9	32.9	5.66	15.4	16.6
LN	43	49.9	52.4	38.8	51.6	38.8	21.6	43	46.8
liver	1.35	2.48	3.38	2.9	3.51	2.16	0.42	1.12	1.69
lung	35	48.1	49.3	40.7	52.8	49.1	31.9	39.1	38.5
spleen	26.4	28	34.2	28.6	31.2	19.5	5.45	22.5	17.8

Supplementray Figure 5d

	αPDL1			coblockade			ctr		
mouse	1	2	3	1	2	3	1	2	3
BM	7.29	7.03	7.271	7.29	7.47	7.69	6.584	7.09	7.76
blood	8.14	8.29	8.889	9.6	8.91	8.91	7.093	8.38	8.82
Kidney	9.44	9.75	10.93	12.2	10.7	10.2	7.958	8.29	8.45
LN	9.24	9.75	10.13	9.47	10.5	9.24	7.869	9.22	8.76
liver	7.12	7.94	7.781	7.43	8.18	7.69	7.204	7.63	7.54
lung	8.73	9.66	9.82	9.33	10.4	10.1	8.778	8.73	8.73
spleen	9.66	8.89	9.598	9.33	9.6	9.13	7.781	7.34	8.38

Supplementray Figure 5e

	αPDL1			coblockade			ctr		
mouse	1	2	3	1	2	3	1	2	3
BM	8505	8794	7333	5533	10530	9906	8392	7939	7663
blood	2013	2593	1771	2588	2136	2177	6502	4453	2029
Kidney	10156	8027	7629	5039	9218	10064	7731	5370	5676
LN	5451	4444	3723	6120	5246	5664	4841	3083	4142
liver	10578	6674	5213	6987	7852	8639	3663	3671	3902
lung	5291	5701	5862	4444	5616	7446	3107	3266	3855
spleen	4913	3959	4057	4575	5474	5093	2319	3214	2843

Supplementray Figure 6a

	blood	spleen	BM	LN	lung	liver	kidney
mouse_1	0.034135	0.039027	0.028099	0.042316	0.102981	0.035546	0.112529
mouse_2	0.004924	0.002074	0.005955	0.008624	0.066489	0.005254	0.044434
mouse_3	0.016498	0.006305	0.0038	0.002541	0.067363	0.025852	0.065929
mouse_4	0.035288	0.061505	0.033969	0.066815	0.116355	0.020569	0.237082

Supplementray Figure 6b

	unpulsed				pulsed			
mouse	1	2	3	4	1	2	3	4
blood	3.7	8.36	10.6	4.73	4.64	9.21	9.89	5.26
spleen	12.2	10.8	6.45	10.2	16.5	19.1	18.7	22.5
BM	2.61	4.19	1.59	3.35	9.61	5.31	5.68	7.58
LN	18.2	20.3	23.1	19	11	24.8	25.3	13.6
lung	28.9	36.6	39.9	32.1	35.9	45.5	47.3	43.8
liver	0.69	1.22	1.69	0.49	2.86	1.7	2.61	1.17
kidney	10.7	13.7	11.1	7.93	6.01	13.8	13.6	5.38

Supplementray Figure 6c

mouse	unpulsed 1	2	3	4	pulsed 1	2	3	4
blood	273	320	336	366	308	335	325	286
spleen	290	306	278	289	406	312	378	392
BM	331	279	278	269	431	324	360	387
LN	317	312	332	347	328	332	335	322
lung	321	331	340	350	454	363	377	413
liver	286	290	287	278	337	317	321	305
kidney	298	312	317	308	324	314	318	366

Supplementray Figure 7b

	killed targets	
	tagets + αPDL-1	targets
lung (effector-like)	0.329227	0.116306
lung (exhausted)	0.400372	-0.00523
liver(exhausted)	0.422723	0.009889
spleen (effector-like)	0.318058	-0.11936
spleen(exhausted)	0.434927	0.166889
spleen(memory-like)	0.536269	0.268346
lung (effector-like)	0.43559	0.164324
lung (exhausted)	0.451627	0.097374
liver(exhausted)	0.455361	0.175558
spleen (effector-like)	0.362652	0.071697
spleen(memory-like)	0.102356	-0.13771
